# Synthetic Control of the Defect Structure and Hierarchical Extra-Large-/Small-Pore Microporosity in Aluminosilicate Zeolite SWY

**DOI:** 10.1021/jacs.3c07873

**Published:** 2023-09-27

**Authors:** Ruxandra G. Chitac, Vladimir L. Zholobenko, Robin S. Fletcher, Emma Softley, Jonathan Bradley, Alvaro Mayoral, Alessandro Turrina, Paul A. Wright

**Affiliations:** † EaStCHEM School of Chemistry, University of St Andrews, St Andrews KY16 9ST, U.K.; ‡ School of Chemical and Physical Sciences, Keele University, Staffordshire ST5 5BG, U.K.; § Johnson Matthey, Catalyst Technologies, Billingham TS23 1LB, U.K.; ∥ Johnson Matthey Technology Centre, Sonning Common RG4 9NH, U.K.; ⊥ Instituto de Nanociencia y Materiales de Aragon (INMA), Spanish National Research Council (CSIC)-University of Zaragoza, 12 Calle de Pedro Cerbuna, Zaragoza 50009, Spain; # Johnson Matthey Technology Centre, Chilton TS23 1LB, U.K.

## Abstract

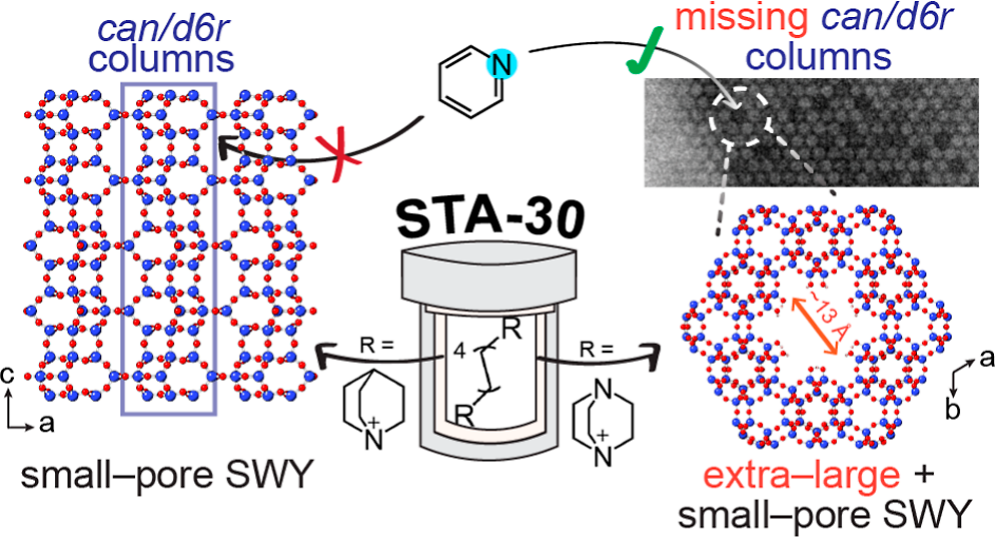

The SWY-type aluminosilicate zeolite, STA-30, has been synthesized via different routes to understand its defect chemistry and solid acidity. The synthetic parameters varied were the gel aging, the Al source, and the organic structure directing agent. All syntheses give crystalline materials with similar Si/Al ratios (6–7) that are stable in the activated K,H-form and closely similar by powder X-ray diffraction. However, they exhibit major differences in the crystal morphology and in their intracrystalline porosity and silanol concentrations. The diDABCO-C8^2+^ (1,1′-(octane-1,8-diyl)bis(1,4-diazabicyclo[2.2.2]octan)-1-ium)-templated STA-30 samples (but not those templated by bisquinuclidinium octane, diQuin-C8^2+^) possess hierarchical microporosity, consisting of noncrystallographic extra-large micropores (13 Å) that connect with the characteristic *swy* and *gme* cages of the SWY structure. This results in pore volumes up to 30% greater than those measured in activated diQuin-C8_STA-30 as well as higher concentrations of silanols and fewer Brønsted acid sites (BASs). The hierarchical porosity is demonstrated by isopentane adsorption and the FTIR of adsorbed pyridine, which shows that up to 77% of the BASs are accessible (remarkable for a zeolite that has a small-pore crystal structure). A structural model of single *can*/*d6r* column vacancies is proposed for the extra-large micropores, which is revealed unambiguously by high-resolution scanning transmission electron microscopy. STA-30 can therefore be prepared as a hierarchically porous zeolite via direct synthesis. The additional noncrystallographic porosity and, subsequently, the amount of SiOHs in the zeolites can be enhanced or strongly reduced by the choice of crystallization conditions.

## Introduction

Zeolites are essential materials in many processes that are crucial to the current needs of our society. Some of the most recent examples are conversion of biomass to olefins,^1^ plastic waste degradation,^2^ NH_3_–SCR for NO_*x*_ abatement,^3^ and a variety of adsorption and separation processes.^4^ Their widespread use has resulted not only in the preparation of many new materials with different topology types but also in the gradual optimization of the properties of zeolites of a given framework type via different syntheses and post-synthetic treatments. Typically, the framework Si/Al ratio, crystal size and morphology, and extra-framework cation content are modified. More recently, the distribution of Al in the framework has attracted attention as an important parameter,^5^ as have two additional properties, silanol content and hierarchical porosity.^6^ The latter two are often associated because mesopores cut through the aluminosilicate framework and are terminated by silanol groups.

**Figure 1:**
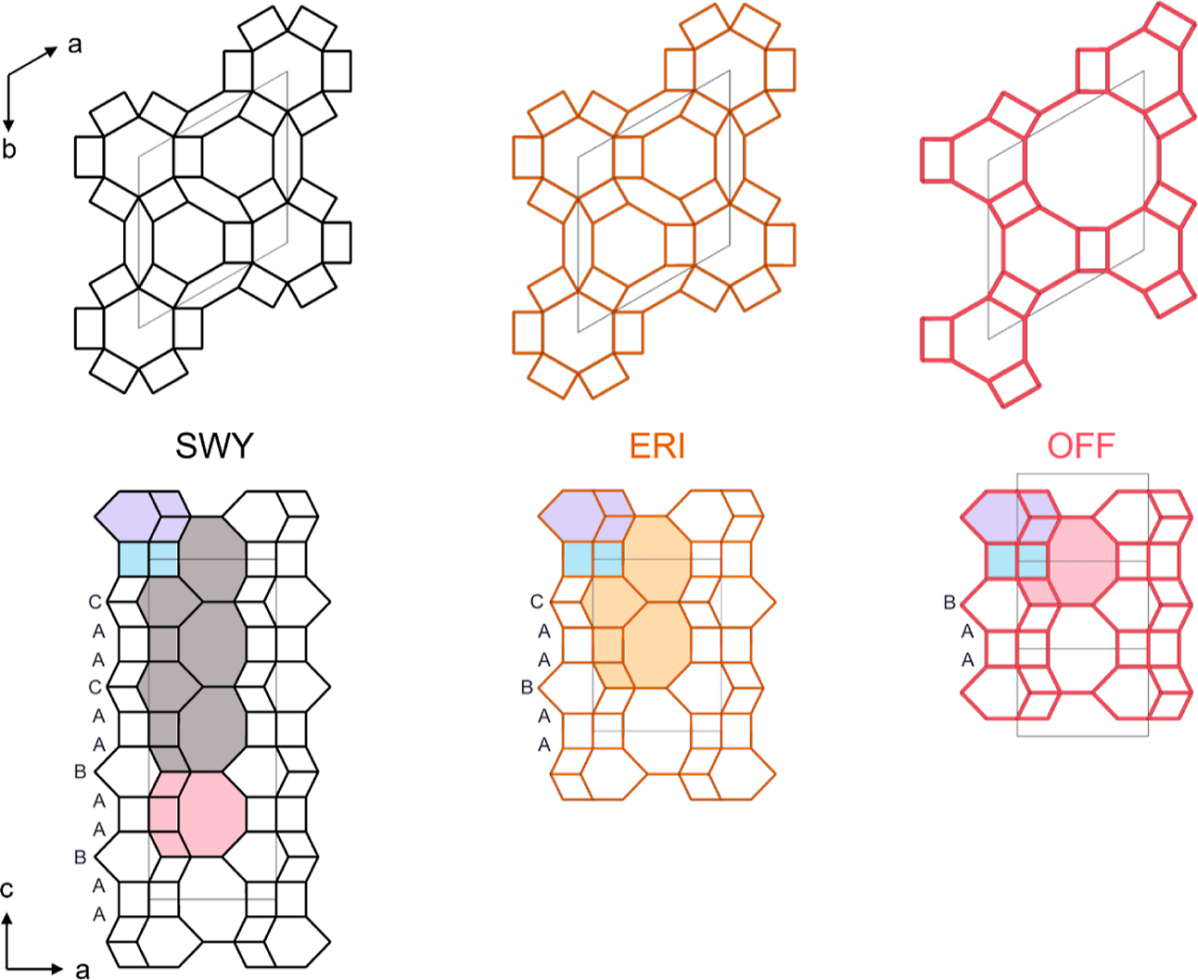
SWY (left), ERI (middle), and OFF (right) topologies presented along the *c*-direction (top) and along the *b*-direction (bottom). The 6-ring stacking sequences are annotated on the structure, and the characteristic cages are shaded in different colors (pink—*gme*, purple—*can*, blue—*d6r*, gray—*swy*, and orange—*eri*).

We recently reported the synthesis of the small pore aluminosilicate zeolite STA-30, structure-type SWY. It is a member of the erionite–offretite family based on columns of cancrinite (*can*) cages and double 6 rings (*d6rs*).^7,8^ (Figure 1) STA-30 was prepared by a designed synthesis, making use of its chemical and structural similarity to erionite (ERI) and employing computational chemistry to model templating of the characteristic elongated *swy* cavity. The organic structure directing agent (OSDA) 1,1′-(octane-1,8-diyl)bis(1,4-diazabicyclo[2.2.2]octan-1-ium) (diDABCO-C8^2+^) was predicted to fit the cavity well and found experimentally to give the most crystalline material, and upon calcination and loading with copper cations, it was found to be active in the NH_3_–SCR reaction.

One characteristic feature of this STA-30 is the very high content of silanol species in the activated K,H-form observed by ^1^H MAS NMR, when compared with the level of Brønsted hydroxyls. The level of hydroxyls is similar to that observed previously for high silica-templated ZSM-5 prepared in alkaline media in the absence of inorganic cations (where the defects act to balance the positive charge of the OSDA)^9^ and in zeolite beta,^10^ where silanols have been inferred to exist around extended cavities where growth defects occur. Silanol defects are of importance in catalytic reactions, either improving the performance, for example, by changing the hydrophilicity^11^ or reducing it, for example, by inducing coking.^6^ Furthermore, they can reduce the hydrothermal stability of the framework.^12^

Therefore, we investigated whether the concentration of the silanol species in STA-30 can be controlled by adjusting the synthesis parameters. In previous studies, for example, Alshafei et al.^13^ showed how modifying the Al and Si sources, the OSDA, and the heating conditions during the synthesis of the related zeolite ERI results in variations of composition and morphology that influence its performance as a catalyst in the methanol-to-olefin (MTO) reaction. Additionally, Palčić et al.^14^ analyzed the crystal morphology and defect sites in MFI-type zeolites and found that the synthetic route is the determining factor in the amount of silanols in the product.

Here, we report on the influence of the source of Al, the added mineralizer, and the OSDA type on the synthesis and resulting properties of STA-30. The synthetic protocols [involving hydrothermal syntheses with or without gel aging and seeding and partial interzeolite conversion (IZC)] have been previously reported by us to give highly crystalline STA-30.^7,8^ Each synthetic variable is analyzed in terms of resulting changes in crystallite morphology and porosity and in the type, concentration, and accessibility of acid sites. This is achieved by complementary X-ray diffraction, electron microscopy, compositional analysis, adsorption, and NMR and IR spectroscopies.

Remarkably, we detected the presence of hierarchical microporosity in STA-30 prepared with 1,4-diazabicyclo[2.2.2]octane-based templates, where access to many of the acid sites of a nominally small pore material is possible via a network of disordered but highly monodisperse extra-large micropores. Hierarchical porosity occurs in zeolites when access to the internal volume of zeolite crystals is possible by a network of pores larger than those defined by their crystalline framework structures.^15^ It is an important attribute of zeolite catalysts because it reduces diffusional pathlengths through the more restricted microporous regions of the zeolite structure and so facilitates molecular transport to those sites in the zeolite that impart their adsorptive and catalytic selectivity.^16^ Here, we report the important role of synthesis conditions in controlling the pore structure, silanol content, and acidity of zeolite STA-30, which are characterized in detail by a combination of analytical methods.

## Experimental Details

### Synthesis

The synthesis procedures of STA-30 are based on published methods.^7,8^ Here, the syntheses differed in the Al source and the alkylammonium additives used as well as the presence or lack of an aging step for the aluminosilicate gel. In each case, the K^+^ levels in the synthesis were adjusted to optimize product crystallinity.

The Al source [aluminum isopropoxide, aluminum hydroxide, or ultrastable zeolite Y (CBV 712)] was dissolved in the mineralizer [an aqueous solution of either 40 wt % tetrapropylammonium hydroxide (TPAOH) or 20 wt % diDABCO-C8 hydroxide] by stirring for at least 30 min at room temperature (RT). If aluminum isopropoxide was used, the solution was heated during mixing to remove the isopropanol formed (monitored by weight difference); the solution was cooled to RT before proceeding. The Si source (Ludox HS-40) was added to this solution, and the resulting gel was stirred for 1.5 h at RT. For samples labeled “TPA”, the gel was aged at 368 K for 20 h before the addition of the SDAs. Solutions of KOH and (OSDA)Br_2_ in H_2_O were prepared and then added dropwise to the aluminosilicate gel. The sample called “TPA_diQuin-C8” used 1,1'-(octane-1,8-diyl)bis(quinuclidin-1-ium) bromide [(diQuin-C8)Br_2_] prepared in-house (Supporting Information) as the OSDA, whereas the other samples were prepared by using (diDABCO-C8)Br_2_. Samples labeled “OSDA” were synthesized using both the hydroxide and the bromide forms of diDABCO-C8^2+^ in their preparation, without using TPAOH as a mineralizer.

**Figure 2:**
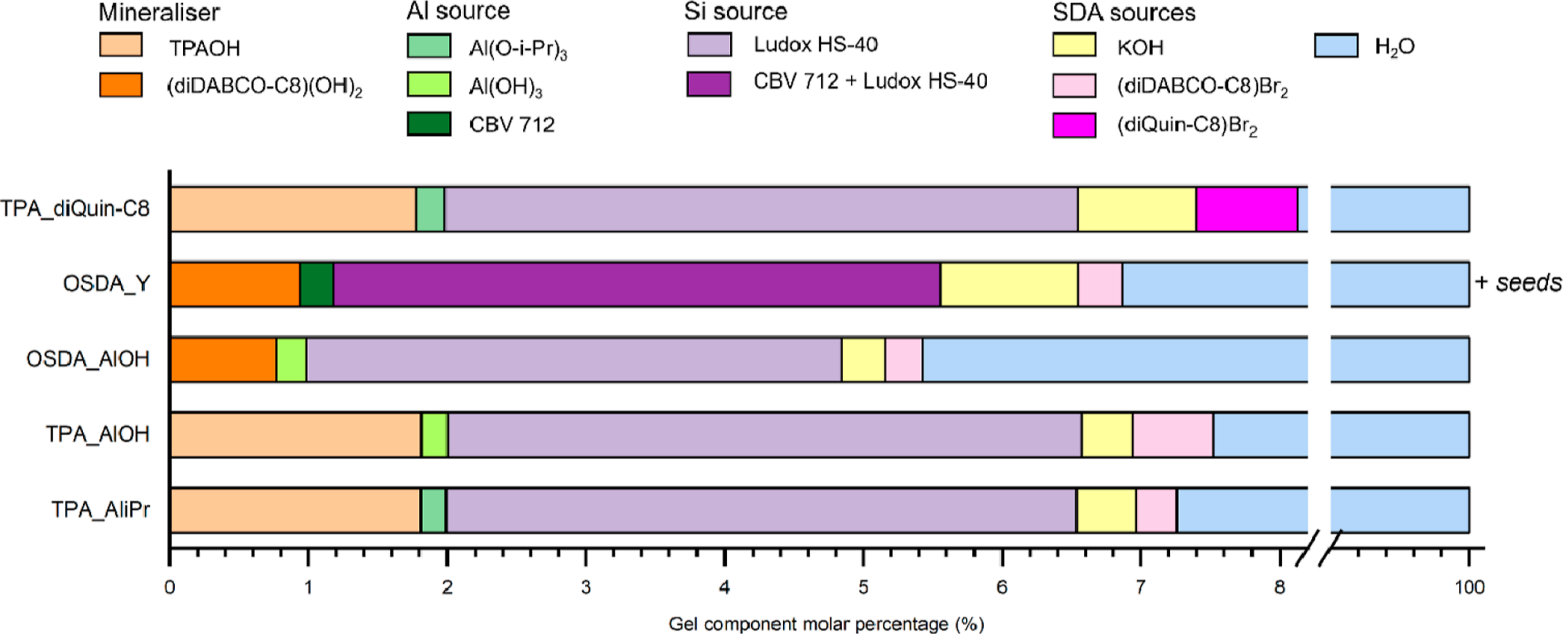
Gel component molar percentages for the syntheses of the different STA-30 materials discussed in this paper (color coded by the reagent source).

The gel was mixed at room temperature for several hours. In the case of the OSDA_Y preparation, seeds of as-made STA-30 were added toward the end of the mixing stage—without this addition another product was formed (Figure S1, Supporting Information). The gel was then heated for several days with constant rotation of the autoclaves. If the synthesis was carried out in large vessels (1.5 L), the gel was instead mixed at 300 rpm using a double-pitched blade type of impeller. The resulting solid products were recovered by centrifugation and subsequently washed with distilled H_2_O until a neutral pH was achieved. The makeup of the gels is presented in Figure 2. The gel compositions, reagents sources, and the specific synthesis conditions can be found in the Supporting Information (Tables S1–S3).

### Ion Exchange and Activation

The as-made samples contained SDAs (K^+^, diDABCO-C8^2+^, or diQuin-C8^2+^), which were removed for characterization of the samples in their “activated” H-form. First, OSDA was removed by heating the samples under a continuous flow of air, up to 823 K for a minimum of 12 h. The calcined samples were then ion-exchanged with 1 M ammonium chloride (NH_4_Cl) solution by stirring them in a proportion of 100 mL solution per 1 g calcined zeolite. This was repeated until all the accessible extra-framework cations were removed, according to X-ray fluorescence spectroscopy (XRF). Then, NH_3_ was removed by another calcination where the sample was heated to 723 K for a minimum of 12 h under a continuous flow of air. The activated samples are denoted with “_H” at the end of their code.

### Analytical Methods

Powder X-ray diffraction (PXRD) was used to analyze the phase purity, crystallinity, and structure of the samples. The patterns were collected in Bragg–Brentano geometry using a Bruker D2 diffractometer equipped with a LynxEye detector (step size of 0.02°, time per step of 48.5 s, at 30 kV and 10 mA using Cu K_α1_ radiation, λ = 1.54060 Å, via a primary monochromator). The sample was rotated at 15 rpm during the data collection to minimize preferred orientation effects.

The amounts of Si, Al, and K in the samples were calculated from XRF spectra collected on a Bruker S8 wavelength-dispersive XRF spectrometer. Thermogravimetric analysis (TGA) and differential thermal analysis (DTA) were used to assess the mass loss in the various samples. This analysis was carried out on a NETZSCH TG1000 M or a NETZSCH STA 449 by heating the sample up to 1073 K at a rate of 5 K min^–1^ under a dry air flow.

The crystallite morphology and size were determined by scanning electron microscopy (SEM). The images were collected on a JEOL JSM-IT800 Schottky field emission scanning electron microscope.

Fourier transform infrared (FTIR) transmittance measurements were performed at about 323 K using self-supported disks of materials dehydrated at 723 K for 5 h in vacuum (with the temperature ramp of 1 K min^–1^). FTIR spectra were collected using a Thermo iS10 spectrometer at a 4 cm^–1^ resolution (0.96 cm^–1^ data spacing). The spectra were analyzed (including subtraction, integration, differentiation, and determination of peak positions) using specialized Thermo-Nicolet software, Omnic 9.3. Accuracy of the maximum positions is estimated to be ±1 cm^–1^. Acidic properties of the activated samples were evaluated using temperature-programmed desorption (TPD) of pyridine (Py), monitored spectroscopically. In brief, an excess of Py was admitted into the transmittance cell at 473 K in a stepwise manner until no changes were observed in the spectra. The saturated sample was then evacuated for 10 min at 473 K to remove physically adsorbed Py, and the FTIR spectrum was collected. The intensity of the Py–H^+^ and Py–L peaks at ∼1545 and 1455 cm^–1^ were used to calculate the concentrations of Brønsted acid sites (BASs) and Lewis acid sites (LASs). The extinction coefficients used in the quantification were those determined for ZSM-5 (aluminosilicate MFI).^17^ Similar experiments were carried out on TPA_AliPr_H with 2,4,6-collidine (2,4,6-trimethylpyridine) at 473 K. For this sample, the acidic properties were also probed with ammonia. In the transmittance TPD experiments, ammonia was removed under vacuum in a stepwise fashion at 423–723 K, and the FTIR spectra were collected every 50 K. The amount of adsorbed ammonia was determined as a function of temperature. All spectra were plotted using Origin software.^18^

Solid-state magic angle spinning (MAS) NMR (SS-NMR) spectra were used to study the OSDA encapsulated in the as-made samples as well as the Al, Si and H environments in the various forms of the materials (as-made, calcined, exchanged, activated). The samples were dried overnight before collection of ^29^Si spectra (at 383 K) and ^1^H spectra (at 573 K and packed under vacuum). For the collection of ^27^Al spectra, the samples were kept in a humid environment overnight prior to measurement. After the appropriate pre-treatment, the samples were packed into MAS rotors. The NMR spectra were acquired at a static magnetic field strength of 9.4 T (ν_0_(^1^H) = 400 MHz) or 14.1 T (ν_0_(^1^H) = 600 MHz) on a Bruker Avance III console using TopSpin 3.1 software or a Bruker Avance Neo console using TopSpin 4.0 software. The rotors were spun using room-temperature-purified compressed air at 14 kHz (^13^C, ^1^H, ^27^Al), 12.5 kHz (^13^C) or 4 kHz (^29^Si). ^13^C spectra were collected using cross-polarization (CP). For ^29^Si, the probe was tuned to 79.49 MHz and referenced to kaolinite at −91.2 ppm. For ^27^Al, the probe was tuned to 156.40 MHz and referenced to YAG at 0.0 ppm. For ^1^H, the probe was tuned to 600.22 MHz and referenced to *d*16-adamantane at 1.73 ppm. For ^13^C, the probe was tuned to 150.93 MHz and referenced to alanine CH_3_ at 20.5 ppm.

Solution NMR spectra of various OSDAs dissolved in D_2_O were collected on either a Bruker AVIII 500 or a Bruker AVII 400 spectrometer.

CHN analysis was carried out on an elemental analyzer model CE-440 by Exeter Analytical Inc.

For transmission electron microscopy studies, materials were crushed using a mortar and pestle and dispersed in ethanol. A few drops of the suspension were placed onto holey carbon copper grids. The measurements were carried out using an FEI Titan XFEG operated at 300 kV. The column was fitted with a CEOS spherical aberration corrector for the electron probe. Prior to the experiments, aberrations were minimized using a gold standard sample, assuring a point resolution of 0.8 Å. Data were collected using an annular dark field detection (ADF) instrument with the inner angle set at 30 mrad and the outer angle set at 180 mrad. Due to the high electron beam sensitivity of the zeolite samples, data acquisition and analysis were performed using the Realtime module and the HREM Filters Pro from HREM Research Inc.^19^ To minimize the beam damage, the electron dose used was maintained at 1500 e^–1^ Å^–1^. Image simulations were performed using the QSTEM software, based on the multislice method.^20^ A supercell of 118.434 × 200.803 × 90.719 Å^3^ with a column of *can* and *d6r* cages removed was constructed for the simulations, introducing the same parameters used experimentally.

The porosity of the activated samples was probed by measuring Ar adsorption isotherms at 87 K and isopentane adsorption isotherms at 293 K using a Micromeritics 3Flex apparatus fitted with a ColdEdge cryostat. The physisorbed water in the samples was removed prior to the measurement by heating them under vacuum at 623 K for 16 h. The isopentane was degassed to remove any dissolved air by using a freeze–thaw method before use. For one of the samples, after the measurement of the Ar adsorption/desorption isotherm at 87 K, the sample was re-outgassed in situ as previously described. The isopentane isotherm was terminated at a relative pressure of 0.36 *p*/*p*_0_. At this point on the isotherm, isopentane occupies any accessible microporosity. The temperature of the cryostat was lowered to 87 K, thereby freezing the adsorbed isopentane within the accessible micropores. The sample was then evacuated overnight to better than 1 × 10^–5^ mmHg, and another Ar isotherm was measured to probe the pore volume accessible only through small pores.

The catalytic cracking of a mixture of two hexane isomers over K,H-STA-30 catalysts was performed according to the method reported by Carpenter et al.^21^ that was originally devised by Frillette et al.^22^ and has been widely used in studies of the acid forms of zeolites.^23–25^ The linear alkane should be able to diffuse through 8R openings in the SWY structure, whereas the branched alkane will not. A 1:1 molar mixture of *n*-hexane/3-methylpentane (Sigma-Aldrich) was prepared and slowly injected via a Nexus 6000 syringe pump into a nitrogen gas stream that was then passed over the zeolite catalyst in a stainless-steel reactor at reaction temperature. The outlet gas was analyzed by a flame ionization detector using an Agilent Technologies 7890B GC fitted with a Porabond Q (25 μm × 320 μm × 5 μm) column, which had been calibrated by passing *n*-hexane/3-methylpentane/N_2_ through an empty reactor. 0.5 g of zeolite powder was pelletized and sieved into a particle size fraction of 0.42–0.84 mm. This catalyst was introduced onto a silica frit in the reactor and activated at 623 K in flowing N_2_ for 14 h prior to cooling to 603 K. The hexane/3-methylpentane mixture was then passed over the catalyst at that temperature at 28 μL (liquid) min^–1^ in 20 mL min^–1^ of a 3:1 N_2_/Ar mixture, giving a liquid hourly space velocity (LHSV) of 1.68 h^–1^ (considering the catalyst bed volume as 1 mL). A few samples were taken at 603 K before the reactor was raised in temperature to 623, 648, 673, and 698 K, the reaction products being followed and quantified throughout. The overall amounts were normalized against the total detected hydrocarbon signal, which showed fluctuations of a few percent.

### Computational Details

All calculations were performed in Materials Studio by BIOVIA,^26^ using either the Forcite or Sorption modules. COMPASS III^27^ was the force field used, and the charges on the framework atoms, extra-framework cations, and Ar atoms were force field-assigned initially. An opposing charge was spread across all of the framework atoms to balance any extra-framework cations present and maintain a neutral unit cell. The empty silica framework structure of SWY was downloaded as a .cif document from the IZA database. It was converted to *P*1 symmetry before any other modifications or calculations.

Geometry optimization and dynamics calculations were used to determine the input model for a unit cell of SWY loaded with K^+^ in all *can* cages (SWY-4K). The unit cell and atomic positions were allowed to optimize during these calculations. The Ewald summation method was used for the calculation of electrostatic terms. The atom-based approach was used for the van der Waals terms, with a cut-off distance of 15.5 Å. The Smart algorithm was used for the geometry optimization and the convergence criteria were Δenergy < 10^–4^ kcal mol^–1^, Δforce < 5 × 10^–3^ kcal mol^–1^ Å^–1^, stress < 5 × 10^–3^ GPa, displacement < 5 × 10^–5^ Å. Convergence was achieved for all calculations in less than 5000 steps. The dynamics calculations were carried out in the NVE ensemble at 650 K. Initial velocities were randomly assigned. The simulations were run with a 1 fs time step, for 50 ps, with structures generated every 500 steps. Consequently, 101 structures were generated as input structures for subsequent geometry optimization calculations. The lowest energy structure was chosen as the input for the adsorption calculations and further editing for generating the supercells with “removed” columns of *can* and *d6r* cages. The supercells (2 × 2 × 1 or 3 × 3 × 1) and removal of the *can/d6r* column was achieved in CrystalMaker.^28^ The edited structures were imported back into the Materials Studio Visualizer to add H atoms to any unsaturated Si–O bonds generated during the removal of the *can*/*d6r* column. Geometry optimization calculations with the same setup as previously described were performed on the modified supercells. The unit cell was allowed to optimize in one set of calculations, but it was kept fixed in another. All calculations achieved convergence in less than 5000 steps, showing that the structures are deemed stable. However, for consistency between the unit cell and supercell calculations in terms of volume, the fixed unit cell geometry optimized 2 × 2 × 1 supercell with a column of *can* and *d6r* cages was used for the adsorption calculations. The smaller size was chosen due to the computational cost.

The adsorption calculations were carried out either as fixed pressure calculations or as an “isotherm”. The Metropolis Monte Carlo method was applied for all calculations.^29^ 10^7^ equilibration steps and 10^8^ production steps were found necessary for achieving C/D ratios of 1.0 across all calculations. The temperature was set at 87 K. The relative probabilities of the exchange, translation, and regrowth steps in the Monte Carlo calculations were 2:1:0.1.

In the case of SWY-4K, two isotherms were measured in the following fugacity ranges: 10^–4^–1 kPa (4 steps), 10^–4^–10 kPa (in 10 steps), and 20–100 kPa (in 8 steps). For the adsorption calculations on the supercell, an isotherm calculation was carried out in the range 10^–4^–1 kPa (4 steps), and three single pressure calculations were also performed at 10, 50, and 100 kPa fugacity values. The average loading was used to quantify the amount of simulated Ar adsorption at any fugacity value.

## Results and Discussion

### Synthesis Routes’ Discussion−Basic Characterization (PXRD, SEM, XRF)

Hydrothermal syntheses with or without aging of the aluminosilicate gel were investigated, and variations in the Al source were also tested to assess whether they have any impact on the final product. Additionally, a partial zeolite interconversion (IZC) route with seeding was also tested. Details are summarized in Table 1.

**Table 1:** STA-30 Sample Codes, Their Synthesis Routes, and Properties of Activated Forms: Si/Al Ratios Determined by XRF; Micropore Volumes Determined by Ar Adsorption; Concentration of BAS and LAS Accessible by Py (Based on the 1700–1400 cm^–1^ Region), as well as Percentage of Total Si–OH–Al Accessible to Py (Based on the 3800–3500 cm^–1^ Region) from FTIR Studies before and after Pyridine Adsorption, as Described in the Text; Data for Activated ERI and OFF Samples Prepared In-House (Details in Supporting Information Figures S2 and S3) Are Provided for Reference. * = Si/Al Calculated by EDS

sample	synthesis approach	Si/Al_XRF_	*V*_micro_ (cm^3^ g^–^^1^)	BAS_Py_ (μmol g^–^^1^)	LAS_Py_ (μmol g^–^^1^)	% total Si–OH–Al accessible to Py
TPA_AliPr_H	hydrothermal, aging	6.0	0.31	305	200	77
TPA_AlOH_H	hydrothermal, aging	7.0	0.31	275	135	68
OSDA_AlOH_H	hydrothermal, no aging	6.7	0.29	180	100	47
OSDA_Y_H	IZC, seeded	6.0	0.27	185	95	46
TPA_diQuin-C8_H	hydrothermal, aging	6.0	0.24	110	85	17
ERI_H	hydrothermal, aging	6.1*	0.25	25	20	7
OFF_H	hydrothermal	3.3*	0.20	285	115	50

In the initial STA-30 synthesis, replicated here as TPA_AlOH, a solution of TPAOH was used as a mineralizer and (diDABCO-C8)Br_2_ was the OSDA salt directing the formation of the *swy* cages.^7^ STA-30 was also prepared using diDABCO-C8^2+^ as both a mineralizer and OSDA (as hydroxyl and salt forms) to rule out any effect of TPA^+^ on the zeolite product. The aluminum sources tested were aluminum isopropoxide, aluminum hydroxide, and zeolite Y (CBV 712). The initial OSDA reported, diDABCO-C8^2+^, was also replaced with diQuin-C8^2+^ (prepared in-house).

**Figure 3:**
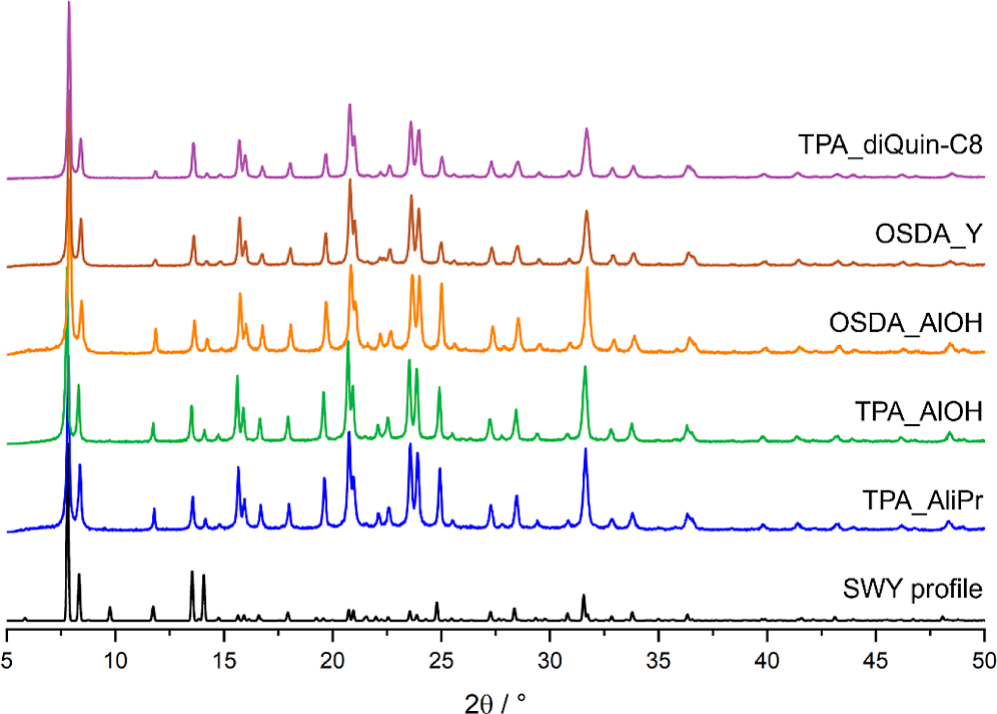
PXRD patterns (using Cu K_α1_ X-radiation) of as-made STA-30 samples prepared through various routes.

Without major changes to the gel composition in terms of the molar content of reagents (Figure 2), zeolite STA-30, aluminosilicate SWY was prepared using all selected synthetic routes, as shown by the PXRD patterns of the as-prepared materials (Figure 3).^7,8^

The advantage of aging the aluminosilicate gels in the “TPA” type syntheses before the addition of SDAs was that full crystallization required much shorter times (2–4 days) compared to the other hydrothermal syntheses (5–7 days). The fastest synthesis was the one using zeolite Y as the Al source in the presence of STA-30 seeds, which required only 24 h for complete crystallization. The PXRD patterns collected during the examination of the crystallization kinetics can be found in Figure S4 (Supporting Information). The faster syntheses of these two approaches can be attributed to the generation of more oligomeric aluminosilicate species in the gel before it is subjected to heat. The ^29^Si NMR data collected from TPAOH and (diDABCO-C8)(OH)_2_ synthesis gels prepared with aluminum isopropoxide and Ludox HS-40 are presented in Figure S5 (Supporting Information), and evidence for the presence of precursor species in the early stages of crystallization in IZC is discussed by Devos et al.^30^ These species then trigger the nucleation phase and subsequently speed up the crystallization phase. Another practical advantage of the TPA syntheses is that all components are in solution, and so it is easy to work with the synthesis mixtures even at a large scale. By contrast, it was more complicated to handle viscous mixtures and additions of solids on a larger scale for the (diDABCO-C8)(OH)_2_-based preparations.

**Figure 4:**
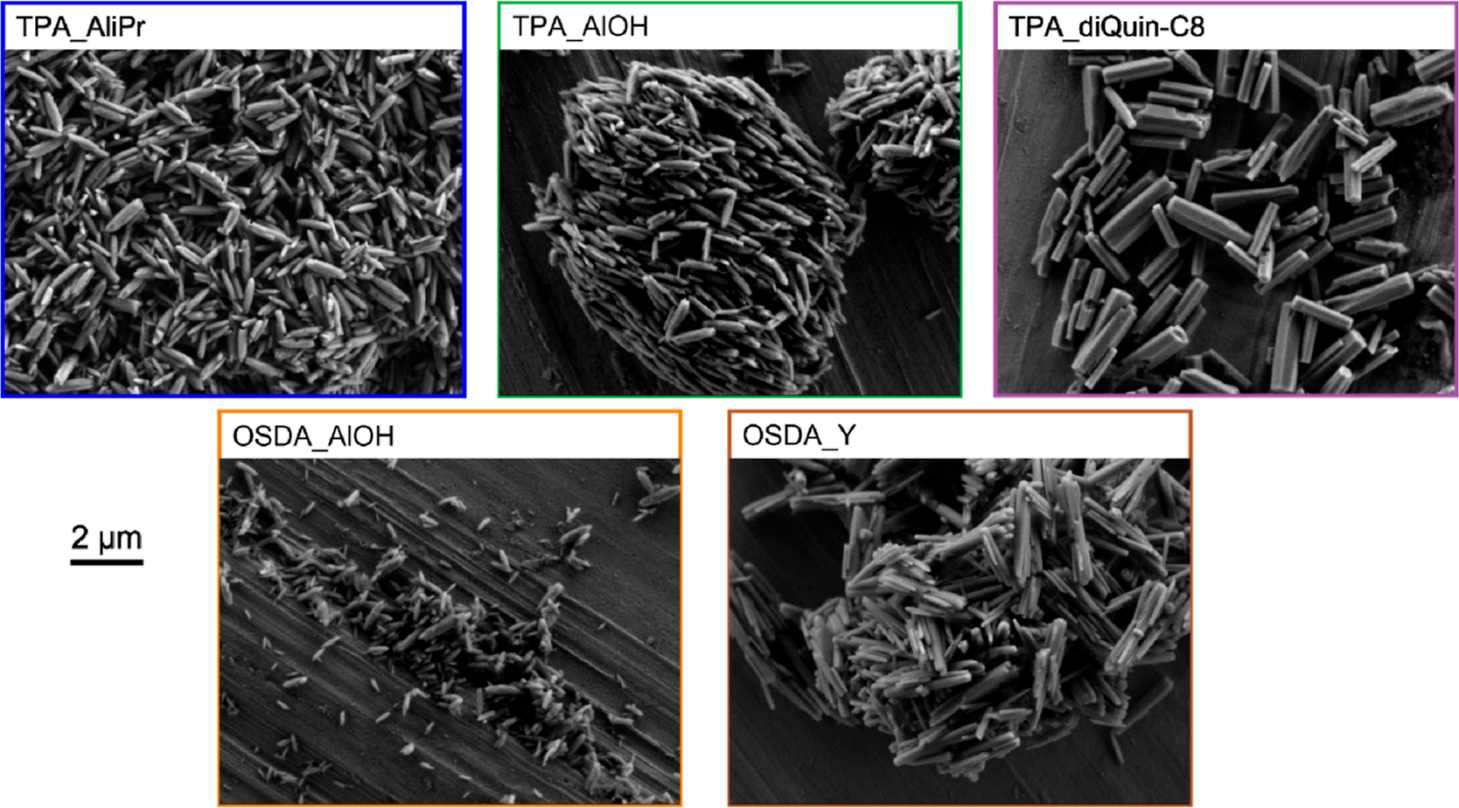
SEM images of STA-30 materials prepared through different routes.

Despite the similarities in the PXRD patterns of the various samples, SEM images revealed variations in the crystallite morphologies among the five samples (Figure 4). While the crystallite morphology of “rice grain” remained consistent for the TPA_AliPr, TPA_AlOH, and OSDA_AlOH samples, the source of the mineralizer caused variations in crystal size. OSDA_AlOH, prepared in a solution of (diDABCO-C8)(OH)_2_, contained crystals in the 0.3–1.0 μm range, while TPA_AliPr and TPA_AlOH, prepared in a TPAOH solution, led to larger crystals (0.8–1.4 μm). Large amounts of organic molecules in the solution have been shown to lead to smaller crystal sizes in other zeotypes,^31,32^ but here, the TPA type gels had larger amounts of organic reagents overall (TPA^+^ + diDABCO-C8^2+^) than the OSDA_AlOH gel. Thus, the decrease in crystal size is likely impacted by the more elongated shape of the diDABCO-C8^2+^ cation that is present in higher quantities in the OSDA_AlOH gel, similar to what has been previously observed in MFI crystals synthesized with TPA^+^ or longer derivatives of TPA^+^.^33^

By contrast, the product of the STA-30 synthesized through partial IZC (OSDA_Y) where commercial zeolite Y, CBV 712 (Si/Al = 6, NH_4_^+^-form), was used as the Al source (and partially the Si source), possessed a crystal morphology that can be described as an agglomeration of matchstick-shaped rods 2–3 μm long and ∼0.5 μm wide. This appears to be a combined effect of the raised concentration of K^+^ in the gel that favors growth in the *c*-direction by promoting *can* cage formation as well as the presence of seeds of STA-30 in the gel that do not seem to fully dissolve at any point during the crystallization (Figure S4, Supporting Information).

A similar effect of the increased K^+^ content giving larger crystal sizes (1.0–2.5 μm) in the *c*-direction can be seen for the sample prepared with diQuin-C8^2+^ as OSDA in TPAOH as the mineralizer. The crystals are hexagonal prisms, a different morphology compared to those observed in the samples prepared with diDABCO-C8^2+^ as OSDA. Hence, in this sample, the organic and inorganic SDAs co-operate to crystallize a zeolite that matches the hexagonal symmetry of the SWY framework.

Si, Al, and K levels in as-made and activated samples were probed by XRF. All Si/Al were similar (Si/Al = 6–7, values in Table 1) which implies that the gel treatment, mineralizer, and Si sources did not strongly affect the Al incorporation. However, the Si/Al of the TPA_AliPr and TPA_AlOH samples showed a difference of ∼1.0 despite having the same levels of Al, Si, K, and mineralizer. Thus, the Al source affected the final Al incorporation in the framework. The effect of Al(OH)_3_ versus Al(O–*i*–Pr)_3_ on the Si/Al_product_ has been previously reported for CHA prepared in HF.^34^ In the context of this study on STA-30, the effect may be attributed to a decreased reactivity of Al(OH)_3_ compared to that of Al(O–*i*–Pr)_3_, which causes a lower incorporation of Al into the aluminosilicate species formed during the nucleation stages and subsequently into the final product. Furthermore, the yield of TPA_AlOH was about one-third that of the TPA_AliPr, further proving the enhanced reactivity of Al(O–*i*–Pr)_3_ under these conditions (Table S1, Supporting Information). The evolution and removal of isopropyl alcohol from Al(O–*i*–Pr)_3_ did not have any effect; products of syntheses without the removal of isopropyl alcohol had the same Si/Al ratio and yields. A relative drop in Si/Al ratio was also observed between OSDA_Y and OSDA_AlOH, the two samples prepared in (diDABCO-C8)(OH)_2_, but this could be attributed to the increased alkalinity of the OSDA_Y gel, which has been shown in the past to have this effect on Al incorporation into the product due to the higher charge density on K^+^.^35^

**Figure 5:**
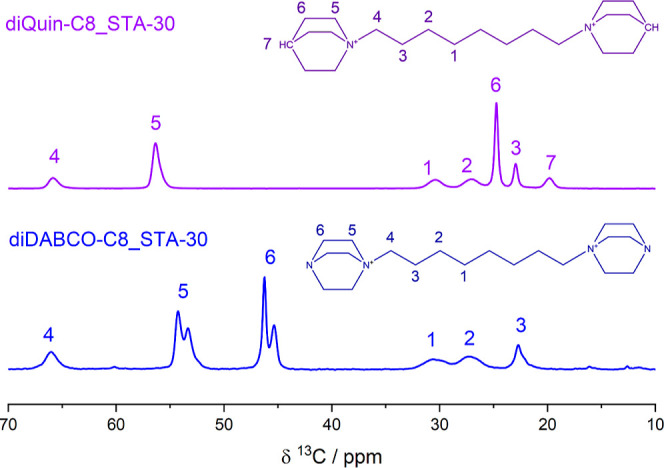
^13^C CP-MAS NMR spectra of diDABCO-C8_STA-30 as-made (bottom, blue) compared with diQuin-C8_STA-30 as-made (top, purple).

To see whether the OSDAs are included intact within the final solids, ^13^C MAS NMR was measured on diDABCO-C8_STA-30 and diQuin-C8_STA-30 (Figure 5). As described previously, the spectrum of diDABCO-C8^2+^ encapsulated in STA-30 was observed as expected, except with splitting of resonances at 54 and 46 ppm, the origin of which was not identified. The spectrum of diQuinC8-STA-30 confirmed that the OSDA was included intact, and no peak splitting was observed for the diQuinC8^2+^. The assignment of peaks to the corresponding environments was based on the NMR spectra of the OSDA bromide salts dissolved in D_2_O (Figure S6, Supporting Information). Note that no splitting of the diDABCO-C8^2+^ peaks was observed in the solution NMR spectrum of the synthesized bromide salt.

In terms of extra-framework cations, K^+^ was present in all samples at similar levels (∼0.6 K/Al in the as-made zeolites), apart from the TPA_diQuin-C8 sample (referred to as diQuin-C8_STA-30 interchangeably), which had ∼0.8 K/Al in the as-made form. This was in line with the TGA data (Figure S7, Supporting Information) that showed a lower mass loss for this sample as well, meaning that less OSDA was present in the diQuin-C8_STA-30 material (13% OSDA in diQuin-C8_STA-30 and 18% in diDABCO-C8_STA-30). Calculations based on XRF, TGA, CHN analysis (Table S4, Supporting Information) and ideal SWY unit cell composition are consistent with 2 OSDA molecules per unit cell for the STA-30 prepared with diQuin-C8^2+^ (K_7.9_(C_22_H_42_N_2_)_2.2_Al_10.5_Si_61.5_O_144_·14.7H_2_O), in line with full occupancy of the *swy* cages. However, similar calculations suggest 3 molecules per unit cell for diDABCO-C8_STA-30 (K_5.9_(C_20_H_40_N_4_)_2.3_Al_10.5_Si_61.5_O_144_·0.9 (C_20_H_40_N_4_), 19.2H_2_O), which is inconsistent with all the OSDAs being in *swy* cages, and requires around one-third of the molecules to be in a different environment (as also suggested by the ^13^C MAS NMR). This is part of an overall picture of the additional porosity that becomes clearer from further analyses.

Similar K^+^ levels were achieved for all samples after activation. Since it has been shown previously that K^+^ can only be removed from the 8MR windows between *swy* and *gme* cages in STA-30,^7^ the remaining K^+^ occupies the *can* cages. Hence, all samples had similar levels of K^+^ in the *can* cages despite differences in synthesis routes, suggesting that the templating of the *can*/*d6r* columns is key for the formation of the SWY topology.

**Figure 6:**
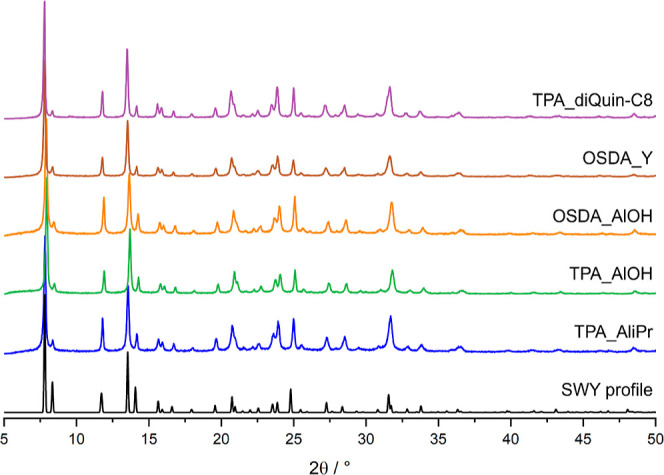
PXRD patterns of activated K,H-STA-30 zeolites (collected with Cu Kα_1_ X-radiation).

Through the activation procedure detailed in the Experimental Section, all STA-30 zeolites were activated without losing crystallinity (Figure 6), which also shows that none of the syntheses routes chosen here negatively impacted the stability of the activated material. The differences in intensity of the peaks are a consequence of the removal of species that occupy the pores and windows of the as-made zeolites.

### Ar Adsorption Isotherms at 87 K—Significant Differences in Uptake

**Figure 7:**
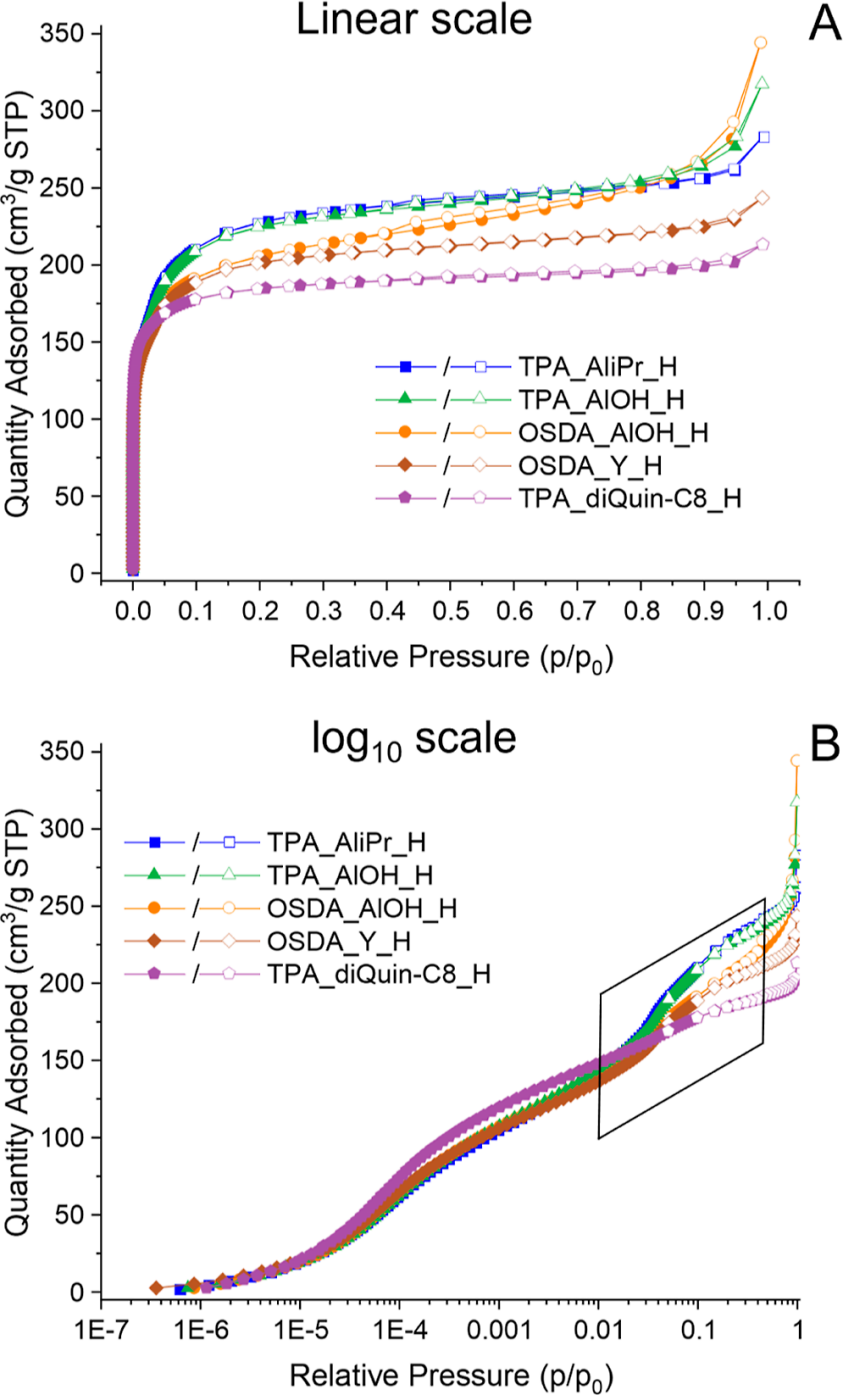
Ar adsorption (filled symbols) and desorption (hollow symbols) isotherms at 87 K measured on activated STA-30 samples. The data are presented on a linear scale (A) and on a logarithmic scale in base 10 (B).

After establishing that all STA-30 zeolites prepared are crystalline and stable in the activated form, we measured their porosity. Ar rather than N_2_ adsorption was used to characterize porosity to avoid effects from the quadrupole moment of N_2_ and so enable accurate analysis of pore size via the Horvath–Kawazoe method. Ar adsorption isotherms at 87 K, collected on activated samples, revealed remarkable differences between the STA-30 materials (Figure 7). The activated STA-30 materials synthesized with diDABCO-C8^2+^ as the OSDA all have higher uptakes, by different amounts, compared to STA-30 prepared using diQuin-C8^2+^. Furthermore, this larger pore volume is associated with a step in their isotherms at around 0.05 *p*/*p*_0_, which is better represented in Figure 7B on the log scale for relative pressure: TPA_diQuin-C8_H shows a typical Type I isotherm in the low relative pressure region with no discernible step.

That there is no step in the sample prepared with diQuin-C8^2+^ indicates that this feature is not due to any phase change or structure change in the Ar adsorbate within the SWY cages, as is observed for some adsorbates in other zeolite structures [such as Ar in silicalite (MFI)^36^]. Instead, these additional features in the isotherms indicate that there is an additional large-pore porosity in diDABCO-C8_STA-30 zeolites.

There are two additional pieces of evidence that show that the pore volume of diDABCO-C8_STA-30 is larger than expected from the crystal structure. The first comes from simulations of the Ar 87 K adsorption isotherm by molecular modeling, as described in the Experimental Section. The uptake at pore filling on the ideal structure (*p*/*p*_0_ = 0.1–0.5) is very similar to that observed experimentally on the diQuin-C8_STA-30 sample, which has a type I isotherm, at ca. 190 cm^3^ (STP) g^–1^ (discussed in more detail below). This agreement suggests that the extra uptake on diDABCO-C8 samples cannot be accounted for by the microporous SWY framework. The other, more indirect evidence is provided by Ar adsorption on an activated form of erionite ERI prepared for comparison (Figure S8, Supporting Information). The specific microporosities of STA-30 and erionite are expected to be very similar, based on the crystal structures, since the pore volume of two *eri* cages in ERI is expected to be similar to the sum of that of the *gme* and *swy* cages present in the equivalent formula unit of STA-30. Indeed, the Ar adsorption of diQuin-C8_STA-30 is very similar to that from erionite (Figure S8, Supporting Information). Extra porosity must then arise from features not accounted for by the crystal structure.

**Figure 8:**
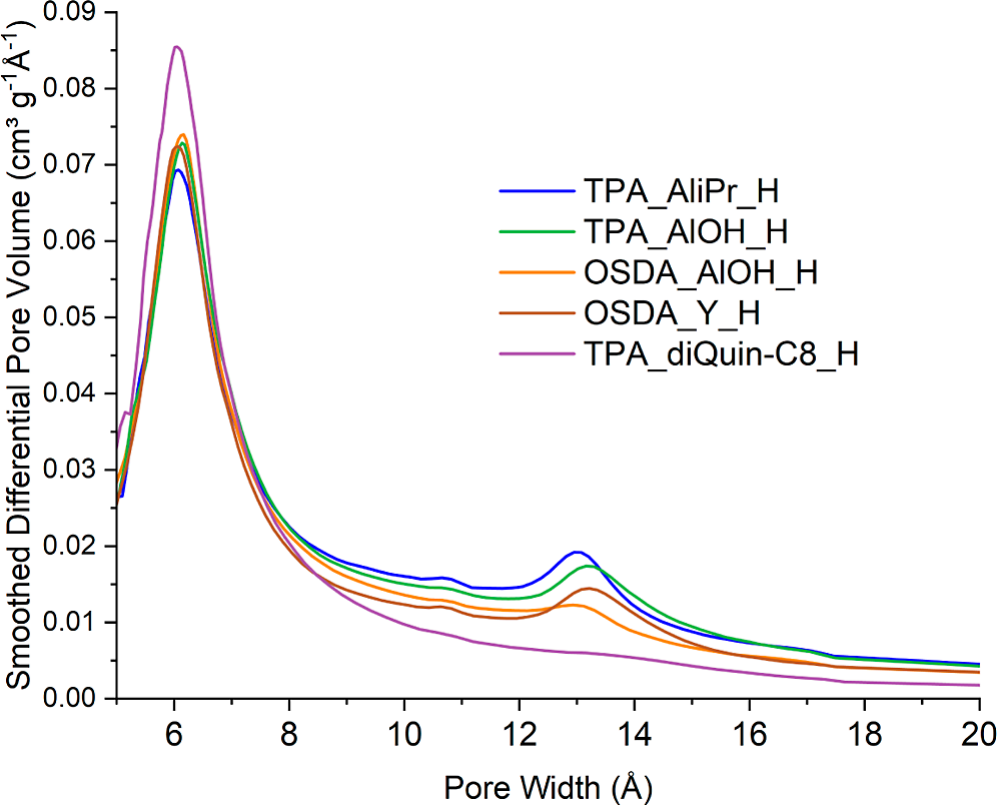
Horvath-Kawazoe plots obtained from Ar adsorption isotherms at 87 K for various STA-30 zeolites.

Horvath–Kawazoe (HK) plots derived from the Ar adsorption isotherms (Figure 8), which plot the effective pore size distribution, show a main peak at around 6 Å for each STA-30 material that can be attributed to uptake within the *swy* and *gme* cages but also an additional peak at 13 Å for the activated diDABCO-C8_STA-30 materials that cannot be explained by the crystal structure. (This feature is barely noticeable in the HK plot of the TPA_diQuin-C8_H.) Therefore, the additional porosity of the diDABCO_STA-30 materials is still in the microporous regime (<20 Å) although at its upper end. Notably, the intensity of the peak around 6–7 Å (from adsorption within the *swy* and *gme* cages) is lower for all diDABCO-C8_STA-30_H materials compared to TPA_diQuin-C8_H, indicating that the additional large pore microporosity, which increases the overall pore volume (from 0.24 to 0.31 cm^3^ g^–1^ in the most marked example), comes at the partial expense of some of the smaller micropores.

Additionally, there is some variation in the isotherms of the diDABCO-C8_STA-30 materials, which correlates with the range of crystal sizes and morphologies observed in the SEM images. While TPA_AliPr_H and TPA_AlOH_H have similar micropore volumes (Table 1), OSDA_AlOH_H has a differently shaped isotherm caused by the greater external surface area and intercrystallite porosity arising from its smaller crystal size. Also, the activated forms of the samples prepared using (diDABCO-C8)(OH)_2_ as a mineralizer have micropore volumes smaller than those of the zeolites prepared using TPAOH.

Based on these observations, significant additional large-pore microporosity can be created in STA-30, the amount of which can be controlled through varying mineralizer and OSDA. In all cases, though, the size of the additional micropores, as measured by the HK plots, remains very similar, and their distributions similarly narrow, which suggests that the pores are associated with a specific structural feature (or more likely with the absence of one). This is discussed in more detail below, after the spectroscopic examination of the defect structure of the STA-30 samples and an investigation of connectivity of the different micropores that is required to establish hierarchical porosity.

### Spectroscopy (SS-NMR and FTIR)—Probing SiOH and Acidity

Ar adsorption data indicate the presence of additional microporosity in STA-30 prepared using diDABCO-C8 as the OSDA compared to SWY zeolites templated by diQuin-C8. Furthermore, TGA and ^13^C NMR data are consistent with diDABCO-C8^2+^ being present in environments other than the *swy* cages. If the extra porosity is associated with framework defects, then these should be visible directly by ^1^H NMR and IR of activated samples, and ^29^Si and ^27^Al NMR could give indirect evidence of their presence. Consequently, all samples were also probed by SS-NMR and FTIR spectroscopies.

**Figure 9:**
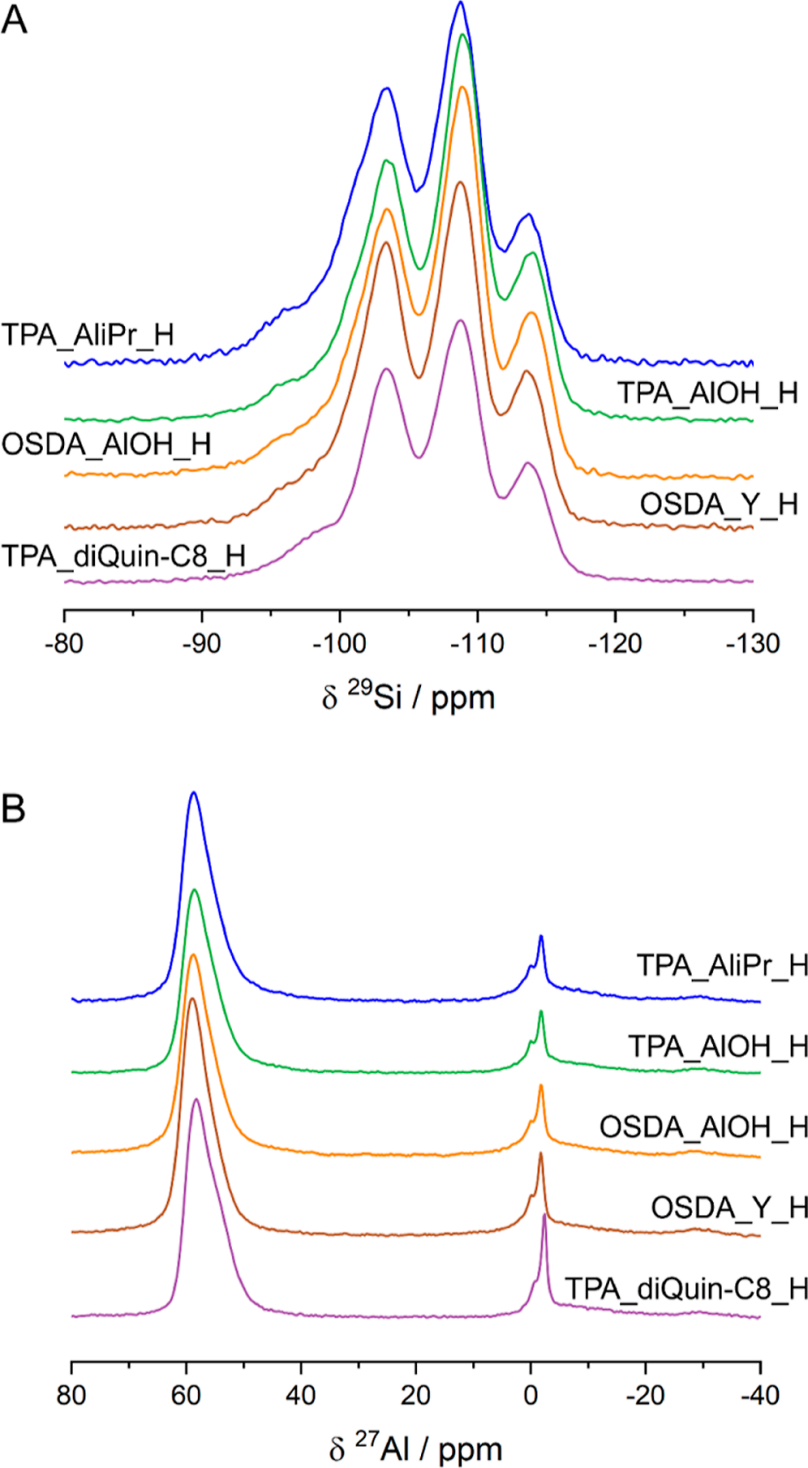
Stacked ^29^Si (A) and ^27^Al (B) SS-NMR spectra of activated STA-30 samples.

The ^29^Si spectra (Figure 9A) show that there are no major differences between the STA-30 samples in terms of the distribution of Al in the framework—the three Si environments identified are Si(OSi)_4_, Si(OSi)_3_(OAl)_1_, and Si(OSi)_2_(OAl)_2_. The SWY topology contains two magnetically distinguishable sites ∼7 ppm apart, among the 3 different T sites (all with multiplicity of 24) in a 2:1 ratio, as previously shown through CASTEP calculations.^7^ Thus, every similar chemical environment leads to 2 peaks in the ^29^Si NMR spectra with intensity ratios of 2:1. For all STA-30 samples studied, deconvoluting the spectra using Si(OSi)_4_ (−108.9, −113.8 ppm), Si(OSi)_3_(OAl)_1_ (−103.5, −108.4 ppm), and Si(OSi)_2_(OAl)_2_ (−96.0, −100.9 ppm) and calculating the Si/Al ratio give reasonable agreement with the values determined from the XRF data (Table S5, Supporting Information). More accurate deconvolution of the diDABCO-C8_ STA-30 spectra would require inclusion of silanol resonances to account for extra intensity and peak broadening observed in the downfield region (−95 to −103 ppm).

The ^27^Al NMR of the activated samples (Figure 9B) reveals that most of the Al remains tetrahedral (58 ppm), although there are two sharp resonances at 0 and −2 ppm, which have been shown to disappear upon ion exchange with NH_4_^+^.^7^ We speculate that these are reversibly coordinated framework-associated octahedral species of the kind proposed in H-mordenite by Ravi et al.^37,38^ A minimal amount of extra-framework Al is created during activation in all samples (broad peak around 0 ppm). The spectrum of the TPA_diQuin-C8_H material exhibits narrower peaks for the octahedral Al environment, which shows that this feature of STA-30 zeolites is not related to the larger micropores but instead is intrinsic to the SWY topology.

**Figure 10:**
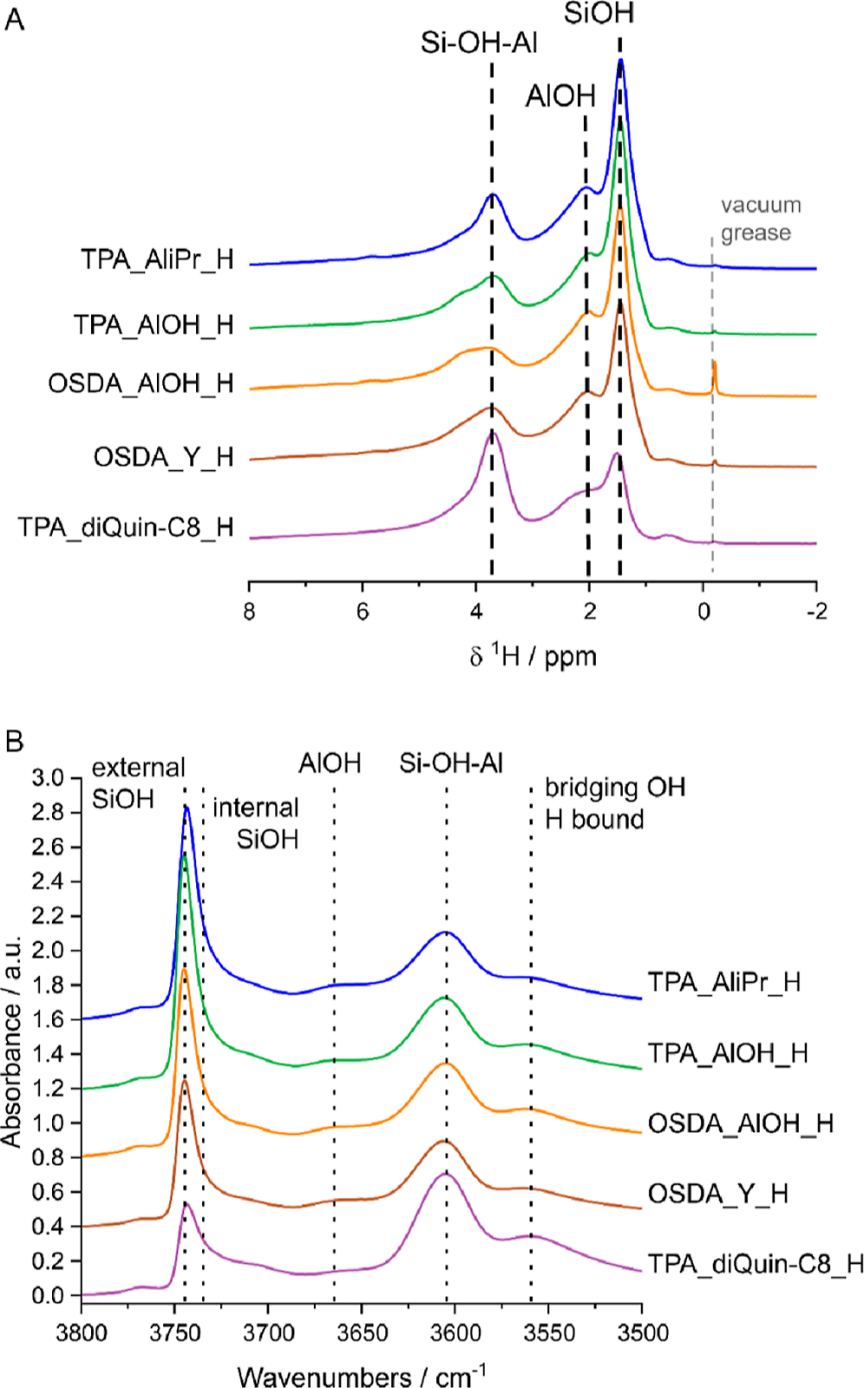
(A) Offset ^1^H NMR spectra of activated samples and (B) offset FTIR spectra of activated samples. FTIR absorbance values were normalized, and spectra were offset for ease of visualization.

Understanding the high level of silanol SiOH observed previously in H,K-STA-30^7^ was a key aim of this study. The ^1^H SS-NMR and FTIR spectra of the dehydrated samples (Figure 10) confirm that all of the zeolites possess a range of hydroxyl groups. Bridging Brønsted acidic OH groups (Si–OH–Al) gives rise to a signal at ∼3.8 ppm in the ^1^H NMR spectra and a band at 3605 cm^–1^ in the FTIR spectra of all activated STA-30 zeolites.^39,40^ These Si–OH–Al groups can be classified as “isolated”, but all samples also contain Si–OH–Al that are H-bonded, based on both the ^1^H NMR spectrum (broad peak at 4.2 ppm) and the FTIR spectra (band at 3560 cm^–1^).^40,41^ AlOH species can be identified in all spectra based on the peak at ∼2 ppm in ^1^H NMR spectra and the band at 3665 cm^–1^ in the FTIR spectra.^41^

NMR and FTIR spectra (Figure 10) show that there is a large concentration of SiOH in all samples prepared with diDABCO-C8^2+^, apparent from the low ratio of the Si–OH–Al/SiOH peak areas. Based on these spectra, TPA_diQuin-C8_H is the only sample that has a Si–OH–Al/SiOH peak area ratio above 1, which means that the concentration of defects, quantified as silanols, was much lower when using diQuin-C8^2+^ as OSDA. Furthermore, the relative ratio of the Si–OH–Al/SiOH increases for those materials prepared with diDABCO-C8 in the order TPA_AliPr_H ≈ TPA_AlOH_H < OSDA_AlOH_H < OSDA_Y_H, showing that the Al source does not play a significant role in the formation of SiOH but that the mineralizer has an impact on the amount of defects present in the activated structures.

These observations are in line with the increase in the maximum uptake in the Ar adsorption isotherms and the consistent differences in silanol concentration between the diDABCO-C8_STA-30 materials and diQuin-C8_STA-30. Thus, it can be concluded that the presence of additional “13 Å” microporosity in STA-30 is linked to an increase in the amount of silanol defects.

To understand the type of acid sites present and their accessibility, the samples were tested further with basic probe molecules. A single experiment investigating the adsorption of NH_3_ onto TPA_AliPr_H showed that all Brønsted sites were accessible to this small molecule, as expected, because it can diffuse through the 8R windows. Upon heating, complete desorption of NH_3_ from BASs was observed by 673 K (Figure S9, Supporting Information). This is in line with what is expected for BASs on small pore zeolites.^42^

The next set of experiments was performed by using pyridine as the basic probe molecule. Pyridine (Py) is used extensively as a probe molecule in FTIR studies because it can interact with SiOH or Si–OH–Al (BAS refers to the sum of these interactions) to form pyridinium cations (Py–H^+^) and it can also coordinate to LASs, such as various Al species in the zeolite.^17,40^ This leads to distinctive bands associated with BASs and LASs. The kinetic diameter of pyridine is 5.4 Å, and so it is too large to pass through 8R windows found in the structures of small pore zeolites, although there may be small amounts of acid sites on the external crystal surfaces and “pore mouths”.^43^ This is demonstrated by measurements on an acid form of the small-pore zeolite erionite, where only a very small fraction of the Si–OH–Al groups observed by FTIR are observed to interact with adsorbed pyridine (Figures S10 and S11, Supporting Information). By contrast, most of the Si–OH–Al hydroxyls observed in the H–form of the large pore zeolite offretite (12R openings) are observed to interact with adsorbed pyridine (Figures S10 and S11, Supporting Information).

**Figure 11:**
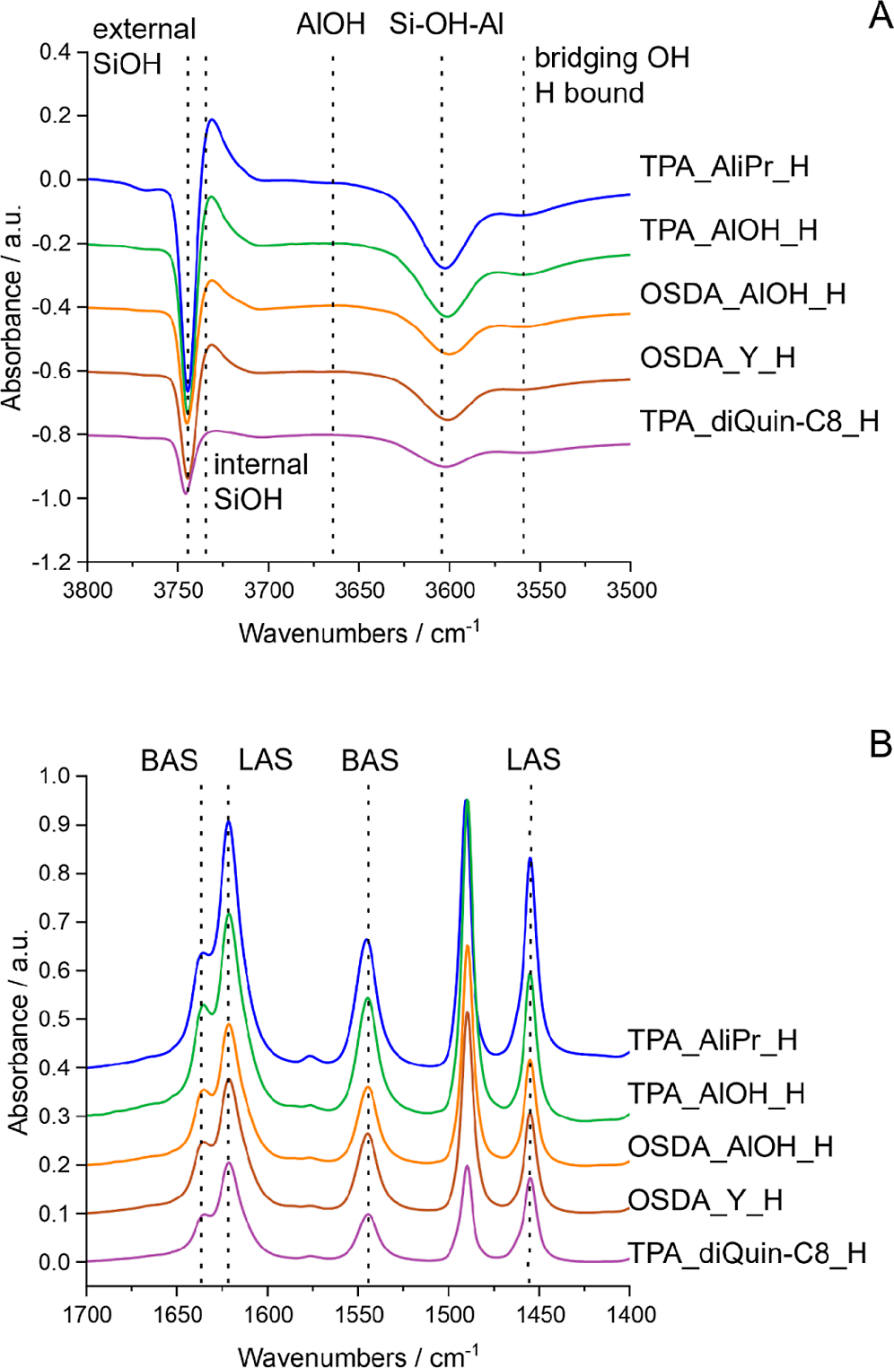
Pyridine accessibility probed by FTIR and presented as difference spectra between the spectrum of the dehydrated samples and the spectrum collected after pyridine adsorbed at 473 K—SiOH region (A) and Py region (B). Absorbance values were normalized, and spectra were offset for ease of visualization.

Remarkably, FTIR difference spectra of the STA-30 samples (which indicate the amount of hydroxyls that interact) show that in addition to many of the silanols being accessible to Py, a large number of the Si–OH–Al and LAS environments are accessible to Py as well, as evidenced by the negative peaks in the SiOH region (3800–3500 cm^–1^) and the peaks in the mid-IR Py region (1700–1400 cm^–1^) (Figure 11). This indicates that access to many of the acid sites of diDABCO-C8_STA-30 materials is possible via rings larger than 8 MR, with accessibility from 46–77% (Table 1). Although pyridine does access significant numbers of acid sites in STA-30 prepared using diQuin-C8, this is a much smaller fraction of the total (17% of the Si–OH–Al) and indicates that this STA-30 is much closer in behavior to the “typical” small pore zeolite erionite (Figure S10, Supporting Information) than are the others.

The high concentration of silanols, and the correlation between their quantity and the additional microporosity (rather than with their crystallite size), suggests that many of them are present in the larger “secondary” micropores and appear at the same frequency as that at which external silanols are usually observed because they are not confined in narrower cages or channels. As an example, while the particle size of OSDA_AlOH_H is much smaller than that of OSDA_Y_H, and the external surface area is consequently much larger (as seen in the Ar isotherm), they have similar levels of silanols and silanol accessibility.

Py-accessible BAS and LAS concentrations measured by observed peaks for pyridinium and coordinated pyridine in the mid-IR show the same trend as the accessibility of SiOH in different STA-30 materials. It is interesting to note that the BAS peak is that of a pyridinium cation (Py–H^+^), which is the result of the interaction of Py with both Si–OH and Si–OH–Al. Since the Py interacts largely with SiOH in TPA_diQuin-C8_H, rather than with Si–OH–Al, it could explain how TPA_diQuin-C8_H still exhibits a high concentration of BAS, even though its Py accessibility to Si–OH–Al is much lower than that of the other samples.

Two additional sets of measurements were carried out to establish the details of the windows giving access to the additional large-pore microporosity: back-exchange of diDABCO-C8^2+^ cations into calcined materials and isopentane adsorption.

### Back-Exchange of OSDAs

**Figure 12:**
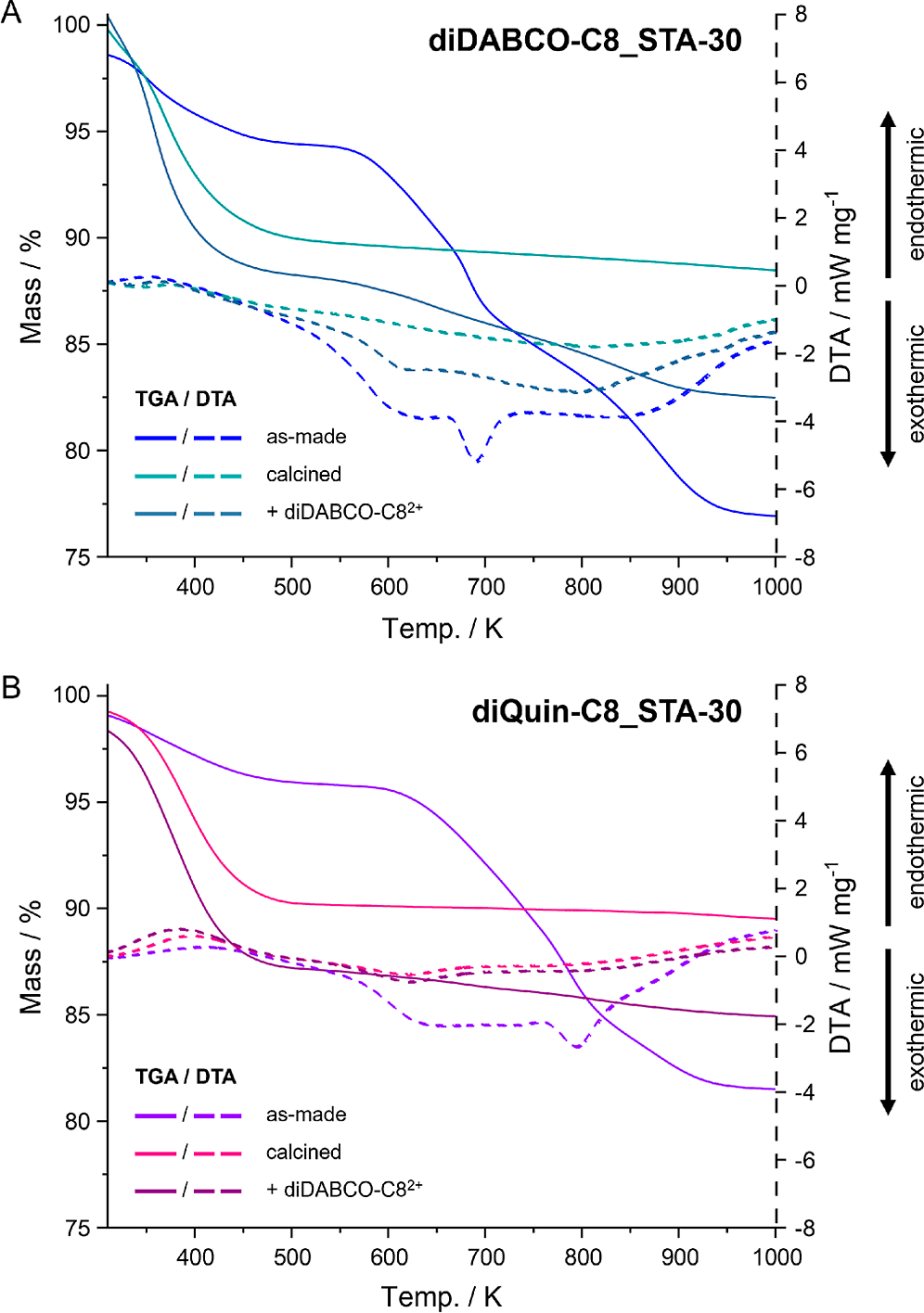
TGA mass loss (solid) and DTA curves (dashed) of as-made, calcined, and diDABCO-C8-exchanged forms of diDABCO-C8_STA-30 (A) and diQuin-C8_STA-30 (B).

To further investigate the porosity in STA-30 samples prepared with either diDABCO-C8^2+^ or diQuin-C8^2+^ as templates, the calcined forms of TPA-type diDABCO-C8_STA-30 and diQuin-C8_STA-30 were stirred in a 10% aqueous solution of (diDABCO-C8)Br_2_. Once the OSDAs are removed from small-pore zeolites by calcination, it should not be possible for them to enter the pore structure because the diDABCO-C8^2+^ cation is too large. Subsequently, TGA of the two “back-exchanged” materials was performed to test whether the OSDA was able to re-enter the calcined zeolites. The results are listed in Figure 12.

For activated diQuin-C8_STA-30 back-exchanged with diDABCO-C8^2+^, there is very little mass loss over the temperature range needed to remove the template from the as-prepared material (<1.5 wt %) and no evidence from the DTA of removal of organic material. The weight loss is therefore attributed to loss of hydroxyls resulting from the repeated mixing with hot aqueous solutions of the OSDA salt. By contrast, the diDABCO-C8 templated solid, after activation and back-exchange with diDABCO-C8^2+^, loses around 5% of sample mass in the 450–900 K range, and the DTA shows a clear exotherm associated with template combustion. Therefore, it is possible to reintroduce ∼1/3 of the initial amount of OSDA in a diDABCO-C8_STA-30 zeolite, but a negligible amount of diDABCO-C8^2+^ can enter its diQuin-C8 counterpart. This is consistent with the STA-30 prepared with diDABCO-C8 possessing additional, extra-large-pore, microporosity that gives access to the small pore structural units via windows that are of medium or large pore size. By contrast, the STA-30 that had been templated by diQuin-C8 behaves like a typical small-pore zeolite.

The re-incorporation of the diDABCO-C8^2+^ was confirmed by the ^13^C CP-MAS NMR spectrum of the back-exchanged sample (Figure S12, Supporting Information). Notably, this spectrum did not show the splitting associated with the DABCO end groups that is observed for the same molecule in the as-prepared material, and the chemical shifts corresponded to the shifts of the smaller components of the split peaks. This indicated that the splitting associated with the DABCO end groups of the OSDA observed in the as-made sample is due to the presence of diDABCO-C8^2+^ in two types of environments—the *swy* cage and the larger micropore and that more (around double) is present in the *swy* cages. By contrast, the ^13^C NMR spectrum of TPA_diQuin-C8 did not show splitting in any of its peaks, confirming the singular role that the diQuin-C8^2+^ cation plays in templating the *swy* cage in STA-30 zeolites.

### Isopentane Adsorption at 293 K

**Figure 13:**
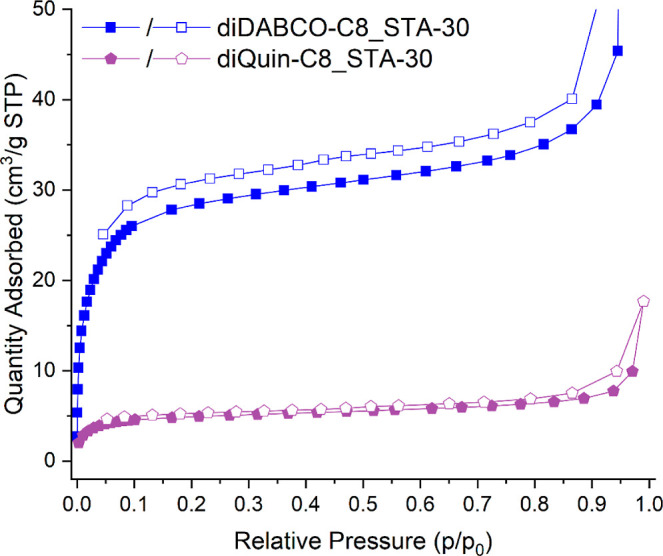
Adsorption/desorption isotherms (full/hollow symbols) of isopentane at 293 K measured on diDABCO-C8_STA-30 (blue) and diQuin-C8_STA-30 (purple).

Finally, adsorption isotherms were collected for isopentane (2-methylbutane) onto diDABCO-C8_STA-30_H and diQuin-C8_STA-30 at 293 K. Branched alkanes such as this cannot pass through the 8R windows, so the uptake of isopentane can occur either on the surface or into the pore space (such as the 13 Å pores observed via the HK plots) accessible via medium or large pore windows. Figure 13 shows that a type I isotherm is obtained for the adsorption of isopentane on diDABCO-C8_STA-30, but there is a very low uptake of isopentane in diQuin-C8_STA-30. The additional pore volume is 0.12 cm^3^ g^–1^ (calculated via the excess uptake at *p*/*p*_0_ = 0.2 and using the liquid density). This again confirms that diDABCO-C8_STA-30 zeolites possess additional porosity above that present in the diQuin-C8_STA-30 zeolites.

In a continuation of this experiment, a sample of STA-30 with additional microporosity was allowed to adsorb isopentane at 293 K at 0.28 bar (*p*/*p*_0_ = 0.36), before being cooled to 87 K to “freeze” the isopentane in place. Subsequent measurement of the Ar adsorption isotherm gave an uptake of 110 cm^3^ (STP) g^–1^ (Figure S13, Supporting Information). This may reasonably be compared to 180 cm^3^ (STP) g^–1^ measured for diQuin-C8_STA-30, although it is plausible that part of the isopentane molecule, while not having full access to the crystalline framework, will take up some 8R window sites at the interface between crystalline and additional porosity. In any case, this demonstrates that most of the crystalline pore space, as defined by the small pore SWY structure, remains accessible even if the larger pores are filled. The HK pore size distribution plot shows that the additional porosity at 13 Å is no longer present when the isopentane occupies these larger micropores. Furthermore, evacuating the sample after this experiment and remeasuring the Ar adsorption at 87 K (Figure S13, Supporting Information) show that both the small and large microporosities can be restored in diDABCO-C8_STA-30 zeolites.

### Model for the Additional Microporosity in diDABCO-C8 STA-30

With the additional porosity confirmed and probed through the studies described above, the next step was to build a structural model to account for these observations. Consideration of the framework structure (Figure 1) indicates that the *can*/*d6r* columns are integral features of the structure, and it is likely that any low energy structural modifications will include them intact, because interrupting either type of small cage to give silanols would be energetically disfavored. Similar columns comprise the framework structures of other zeolites, such as erionite and offretite (Figure 1) and also zeolite L.^44^ Pertinent to the current study, in zeolite T (a random intergrowth of erionite and offretite), atomic force microscopy (AFM) indicates that crystal surfaces are terminated by rows of complete *can* cages^45^ and in zeolite L, careful AFM and HRTEM reveal intact *can*/*d6r* columns on crystal surfaces that terminate as *d6r*s.^46,47^ Additionally, in zeolite L, “columnar nanodefects” are observed by HRTEM at low abundance, in which clusters of *can*/*d6r* columns are occasionally observed to be absent. These columnar nanodefect clusters are of many different sizes, although there is no evidence that single *can*/*d6r* column vacancies occur in the zeolite L studied.^48^

**Figure 14:**
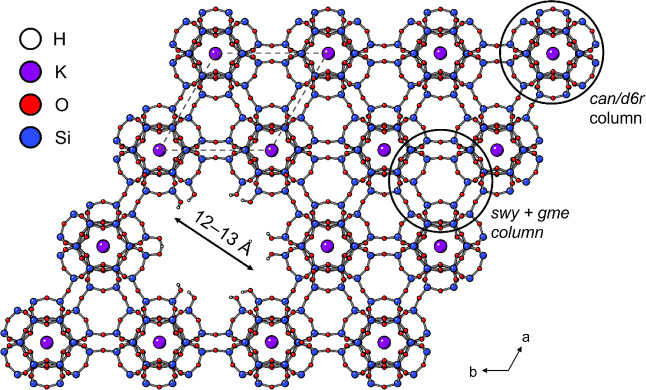
Model for hierarchical microporosity in the SWY framework shown on a 3 × 3 × 1 supercell for ease of visualization; the SWY unit cell size is delineated with a dotted line.

**Figure 15:**
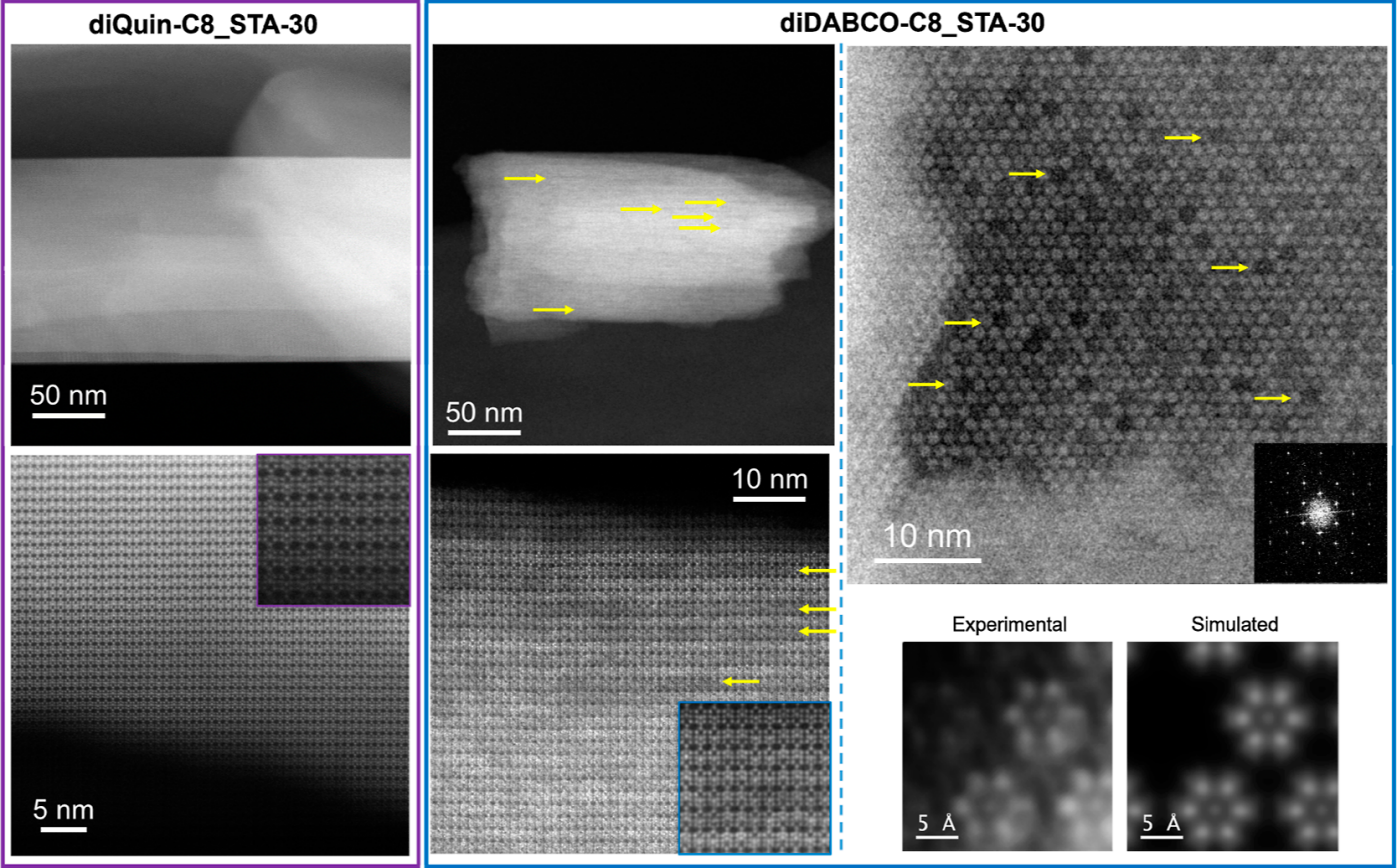
C_s_-corrected STEM–ADF images of diQuin-C8_STA-30 (left, purple outline, down [100]) and diDABCO-C8_STA-30 (middle and right, blue outline, down [100] and [001], respectively), highlighting the differences in contrast in the image of diDABCO-C8_STA-30 that result from the missing *can/d6r* columns (some of these vacancies are indicated by yellow arrows). In the bottom right, a comparison of experimental images of *can* and *d6r* columns is observed in diDABCO-C8_STA-30 down [001] and the corresponding QSTEM simulation of STA-30 with removed *can/d6r* columns and K^+^ occupying the *can* cages.

We therefore considered that columnar nanodefects of a similar kind (missing *can/d6r* columns) could explain the additional microporosity in diDABCO-C8_STA-30. The adsorption data indicate there is a very narrow pore-size distribution, and consideration of the structure indicates that single column defects would be in the correct pore size regime (Figure 14). Furthermore, examination of spherical aberration-corrected (C_s_—corrected) STEM–ADF micrographs of diDABCO-C8_STA-30 and diQuin-C8_STA-30 support this model (Figure 15).

DiQuin-C8_STA-30 has regular morphology with smooth surfaces, and high magnification [100] images indicate uniform contrast over the projection of all *can/d6r* columns. A closer inspection of 6R-layer stacking (inset in the bottom left corner of Figure 15) reveals a very good crystallinity and perfect connection of the *can/d6r* cages. By contrast, STA-30 prepared with diDABCO-C8^2+^ gives crystals with more ragged shapes, and closer inspection of the framework along [100] reveals a difference in contrast consistent with missing single columns, i.e., single rows of *can/d6r* columns in projection which are less bright, indicated by yellow arrows in the images in the middle of Figure 15. Further image analysis is depicted in Figure S15 (Supporting Information), where the models with vacancies along [100] and [001], the simulated data along these two projections, and additional experimental data are presented (also in Figure S16). A closer look (inset in the middle bottom image in Figure 15) reveals the perfect connection among all units, with no structural defects in the form of intergrowth or stacking faults, corroborating the excellent crystallinity of diDABCO-C8_STA-30. Thus, such contrast variations are associated with a change in the thickness of the material (due to missing *can/d6r* columns).

Further confirmation of the presence of extra-large micropores can in principle be accomplished by collecting data along the [001] zone axis; however, the acquisition of high-resolution images perpendicular to the *c*-direction of the crystal is challenging from a technical point of view due to the elongated shape of the crystal. Nevertheless, C_s_—corrected STEM–ADF data were obtained for the diDABCO-C8_STA-30 along [001], see Figure 15 (right). The associated Fast Fourier Transform (FFT) (inset, top right image in Figure 15) is formed by discrete spots, which is an indicator of good crystallinity and is as expected from a well-ordered STA-30 crystal structure. The significant contrast differences observed in the image (examples of which are indicated by yellow arrows in the figure) belong to extra-large pores, revealing the mechanism of formation of the significant additional porosity. Atomic-resolution data (bottom right corner of Figure 15) allow the visualization in projection of the *d6r* units of *can/d6r* columns, where the signal inside the 6Rs correspond to the K^+^ cations within the *can* cages. Simulated data are also presented, based on the model displayed in Figure 14, which is consistent with missing *can/d6r* columns being the origin of the variation in contrast seen in the experimental STEM images obtained.

**Figure 16:**
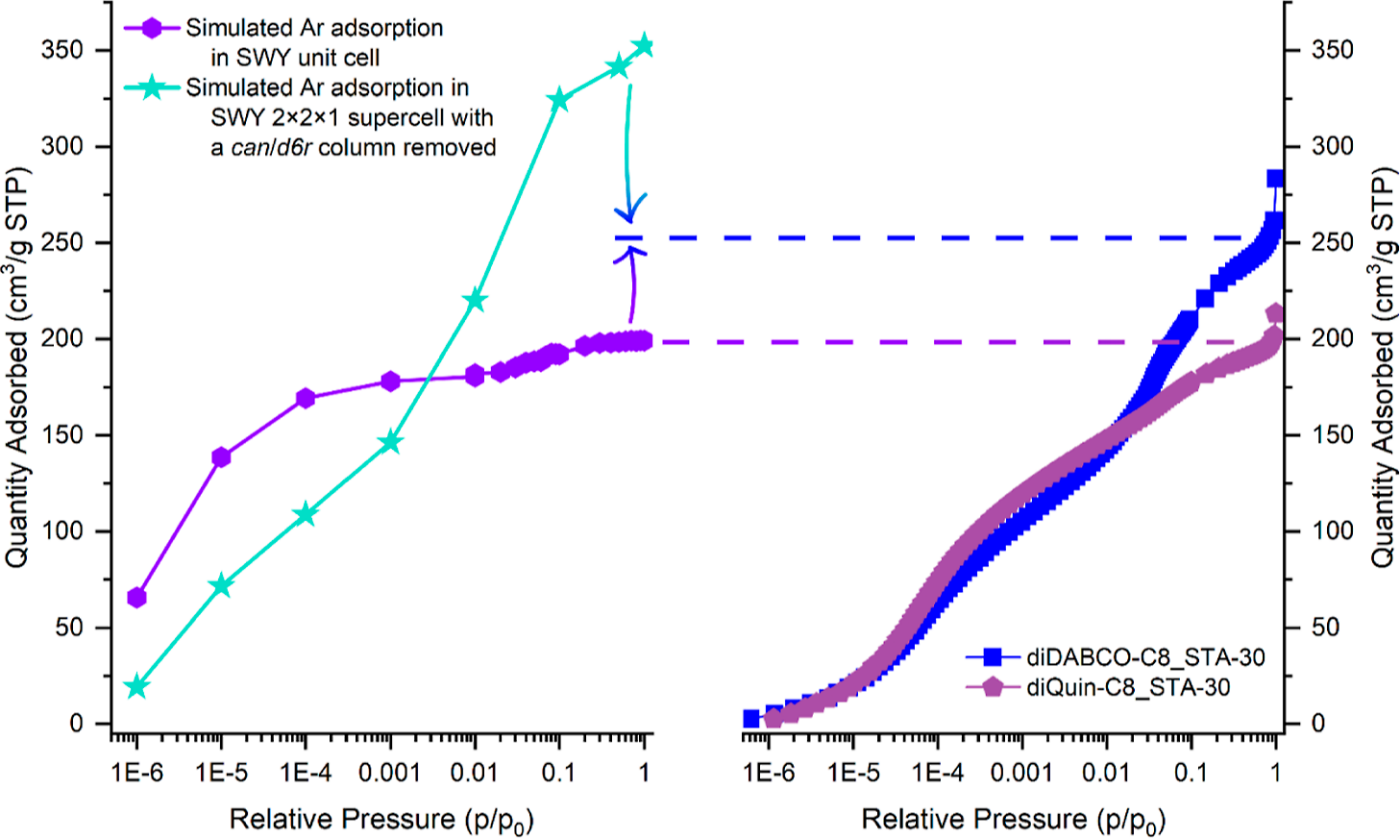
(Left) Calculated Ar adsorption isotherms at 87 K for the ideal SWY structure (purple hexagons) and for a 2 × 2 × 1 SWY supercell with a column of *can/d6r* cages removed (green stars), compared with (right) experimental Ar adsorption isotherms at 87 K measured on diDABCO-C8_STA-30 (blue squares) and diQuin-C8_STA-30 (purple pentagons). The dotted lines show the relations between simulated and experimental data, and the gradient arrows show that both models contribute to the prediction of the uptake in diDABCO-C8_STA-30 zeolites.

To investigate the plausibility of this structural model as the origin of the additional porosity, the STA-30 structure with K^+^ filling the *can* cages was chosen as the parent because it resembled the final activated forms in this study. The Ar adsorption isotherm at 87 K between 10^–4^ and 100 kPa was calculated on an ideal unit cell of this SWY using Materials Studio,^26^ and the simulated uptake closely matched the Ar adsorption isotherm of TPA_diQuin-C8_H (Figure 16).

To simulate the Ar adsorption associated with our model of the single column defect, a structure was constructed in which a complete column of *can* and *d6r* cages in a 2 × 2 × 1 SWY supercell was removed (in CrystalMaker^28^). The addition of H atoms on dangling Si–O bonds within the extra-large pore to give a high concentration of silanols is consistent with the high measured contents of silanol groups by NMR and IR. A geometry optimization calculation was performed on the modeled supercell in Materials Studio using the COMPASS III force field.^26,27^ Convergence was achieved despite the removal of the *can/d6r* column, showing that it is feasible that this could occur inside the zeolite with the retention of overall structural stability.

The Ar adsorption isotherm at 87 K between 10^–4^ and 100 kPa fugacity was calculated at 10^–4^–1 kPa (4 steps), 10, 50 and 100 kPa fugacity on a simulated 2 × 2 × 1 supercell with a column of *can* and *d6r* cages removed from the supercell as previously described. An isotherm with the same step sizes as for the ideal structure was not calculated for this system, because its size incurs large computational costs for each fugacity data point. The simulated values for argon uptake at 50 kPa are ∼40 Ar atoms in the SWY unit cell and 210 Ar atoms in the altered 2 × 2 × 1 supercell. A fugacity of 50 kPa was chosen as the pressure point of reference because at that fugacity there was a very close match between the calculated and experimental uptake of TPA_diQuin-C8_H. The computational models do not exactly reproduce the real system, and so there is some offset between the experimental and calculated isotherms on the pressure axis, but since the uptake remained at a constant value after 40 kPa, it was considered that the value at 50 kPa would provide an accurate average value. Furthermore, all of the experimental Ar adsorption isotherms showed maximum uptake when the 0.5 *p*/*p*_0_ relative pressure was reached, but at higher relative pressures effects such as intercrystallite adsorption could influence the uptake values.

With the aid of these simulated uptakes, it was possible to estimate the frequency of missing *can/d6r* columns that are required for the zeolite to show the Ar uptake experimentally measured on the diDABCO-C8_STA-30 samples (calculations in the Supporting Information). According to this approach, approximately 1 in 10 and 1 in 30 *can*/*d6r* columns are missing from the TPA_AliPr_H/TPA_AlOH_H samples and the OSDA_AlOH_H/OSDA_Y_H samples, respectively. Such missing column defects must be arranged randomly because the crystalline SWY structure is well matched by Rietveld refinements of the structure.^7^

The measurements described above confirm the presence of 13 Å pores in diDABCO-C8_STA-30 consistent with a model in which an SWY framework possesses missing *can/d6r* column defects at a level of up to 1 in 10 (amount dependent on the synthetic route). The additional extra-large pores must be distributed randomly throughout the STA-30 crystals; so to establish whether the porosity of this material is hierarchical, it must be shown that connectivity between these additional pores is possible for molecules larger than 4 Å, the size of the 8R windows that restrict access to the *swy* and *gme* cages of SWY. This has been achieved by isopentane adsorption and OSDA back-exchange experiments. The random distribution of missing column defects suggests that adjacent columns will be missing in some places. Examples can be seen in the STEM image down [001] in Figure 15. This would lead to openings between extra-large pore channels of about 8 Å (Figure S15D). From a catalytic viewpoint, the accessibility of BASs and LASs to molecules larger than 6 Å has been demonstrated by the proven interaction of pyridine and, to a lesser extent, collidine (Figure S14, Supporting Information), with many of the acid sites. For example, pyridine interacts with nearly 80% of the BAS to give pyridinium ions.

The direct synthesis of a hierarchically porous aluminosilicate with extra-large micropores leading to the intact zeolite framework has not previously been reported. Usually, hierarchical porosity in zeolites is considered to result from a combination of mesopores and micropores, where a wide range of routes (direct synthesis or postsynthesis) have been developed for generation of the mesopores.^15,49–52^ Hierarchical porosity has been shown to extend zeolite catalyst lifetime in reactions where coke formation results in pore blocking by providing additional diffusion pathways. This has been shown to occur for the small pore SAPO-34 in the methanol-to-olefins reaction, for example.^53^

It is informative here to consider the structure and Ar adsorption properties of the interrupted germanosilicate zeotype ITQ-43 for comparison.^54^ Its framework structure is fully ordered but possesses many silanol groups that surround extra-large pore channels that extend along the *c* axis and intersect large pore channels bounded by 12Rs along the *a* axis. As a result, Ar isotherms show an inflection at approximately *p*/*p*_0_ = 0.05, and the associated HK plots show the presence of pores with an average diameter of 12 Å, in addition to those at 7 Å expected for the large pore channels. The authors describe this as a hierarchical meso–microporous material on the basis that one dimension of the extra-large pores approaches 20 Å. On the basis of the HK plot, we consider this could be described more realistically as a hierarchical extra-large pore-large pore microporous solid, where our diDABCO-C8_STA-30 material is a hierarchical extra-large pore-small pore microporous material. The structures of large pore STA-30 and ITQ-43 are compared in Figures S17 and S18 (Supporting Information).

Synthesis parameters influence the generation of hierarchical microporosity. The mineralizer source was shown to have some effect on the amount of additional microporosity introduced. Large quaternary ammonium cations have been shown to introduce hierarchical mesoporosity in materials such as ZSM-5 (MFI).^55^ Here, the presence of TPA^+^ in the gel could serve as an initiator for the formation of larger microporosity as it might hold aluminosilicate precursors further apart in the solution due to its larger radius and lower charge density compared to diDABCO-C8^2+^. However, it must act in concert with diDABCO-C8^2+^ cations and enhance their effect.

Most significantly, however, the diDABCO-C8^2+^ molecules are the principal cause of hierarchical microporosity in STA-30. They serve a dual structure directing purpose, as confirmed by ^13^C CP-MAS NMR (Figure 5). Thus, diDABCO-based templates act in a similar manner to soft templates which are used in hierarchical mesoporous zeolites to template specific cages in the target zeolites as well as to act as the mesoporogen.^15^ The dual role of the diDABCO-C8^2+^ template is enabled because the width of the pore created by the removal of the *can/d6r* columns matches the width of the OSDA molecule. Meanwhile, diQuin-based OSDA acts only as the template for the *swy* cage, as evidenced by the lack of splitting in any of the peaks in its ^13^C CP-MAS NMR: it is not capable of structurally directing the formation of the larger micropores. The most relevant difference between these two molecules is the fact that the DABCO unit terminates with an N atom with a lone pair of electrons, whereas the quinuclidine unit terminates with a C–H group. We speculate that the lone pair enables the DABCO end-group to H-bond to a Si–OH of a *can/d6r* column during crystallization and so disrupt the growth of the *can/d6r* columns of the zeolite. Furthermore, once this larger micropore might start to form due to this interaction, diDABCO-C8^2+^ molecules could template the larger micropore by fitting across the pore, normal to the *c*-direction. The H-bonding of some of the DABCO units would then result in a set of environments different from those of the molecule that templates the *swy* cage, as observed in the ^13^C NMR spectra of the as-made and diDABCO-C8^2+^-loaded diDABCO-C8_STA-30 materials. Since the C–H group on the quinuclidine end group of diQuin-C8^2+^ is not capable of forming H-bonds with Si–OH, it would not be expected to favor the interaction between the OSDA and the framework, so it does not lead to the formation of the additional micropore. More generally, we speculate that another OSDA with an available lone pair on a terminal N that could form H-bonds could replace diDABCO-C8 in this synthesis if it is a good fit to the *swy* cage and the extra-large pore.

### Catalytic Cracking of *n*-Hexane and 3-Methylpentane

To gain more information about the pore structure and the accessibility of acid sites, the catalytic cracking of a mixture of *n*-hexane and 3-methylpentane was performed over samples of STA-30 prepared with (diQuin-C8)Br_2_ and (diDABCO-C8)Br_2_. This reaction is used to determine the effective pore size of zeolites.^21–25^ Different authors have discussed the limitations of this method, but all agree that while the acid forms of small pore zeolites convert almost no branched alkanes, large or extra-large pore zeolite solid acids convert similar amounts of the isomers. The cracking of the 1:1 mixture of *n*-hexane and 3-methylpentane was performed as the temperature was increased from 603 to 698 K. Estimated conversions of the *n*-hexane and 3-methylpentane are given in Table 2. Due to fluctuations in total measured signal, these data should be taken as indicative.

**Table 2:** Catalytic Conversions and Ratios of Conversion of *n*-Hexane (*n*-hex) and 3-Methylpentane (3-mp) at Various Temperatures over K,H-STA-30 Prepared with diDABCO-C8^2+^ or diQuin-C8^2+^^a^

temp./K	diDABCO-C8_STA-30_H			diQuin-C8_STA-30_H		
	*n*-hex conversion/%	3-mp conversion/%	conversion ratio	*n*-hex conversion/%	3-mp conversion/%	conversion ratio
603	30(3)	8(3)	4	62(3)	0(3)	>20
603	20(3)	5(3)	4	30(4)	8(4)	4
603	17(3)	5(3)	3	25(3)	3(3)	8
623	18(3)	5(3)	4	27(3)	4(3)	7
623	15(3)	5(3)	3	20(3)	4(3)	5
648	20(3)	6(3)	3	24(3)	5(3)	5
648	18(3)	6(3)	3	20(3)	5(3)	4
673	21(3)	8(3)	2	20(3)	5(3)	4
673	n.d.	n.d.	n.d.	17(3)	5(3)	3
698	24(3)	11(3)	2	18(3)	5(3)	4

a A 1:1 mixture was passed over 0.5 g of the catalysts with an LHSV of 1.68 h^–1^, in a stream of 20 mL min^–1^ N_2_/Ar (3:1); (n.d. = not determined).

The data show that diQuin-C8_STA-30_H is more active for *n*-hexane cracking, particularly in the earlier stages of the catalytic test, which is consistent with this sample possessing more BAS sites and fewer silanols. Furthermore, in both samples, considerably more *n*-hexane is converted than 3-methylpentane, indicating that most of the catalytic conversion occurs at BASs within cages of the SWY crystal structure in diDABCO-C8_STA-30_H rather than in any extra-large micropores, where the conversion rates of the isomers would be similar.

There is also a strong indication that diQuin-C8_STA-30_H is the more selective of the two zeolites for cracking of the linear alkane, which shows that mainly linear molecules have access to the active sites in this material, as expected from the 8R window openings. For diDABCO-C8_STA-30_H, this preference is less marked, suggesting that more of the branched isomers have access to acid sites, in this case accessible from the extra-large pores. A more detailed catalytic study is required to quantify these trends.

## Conclusions

The zeolite STA-30 (SWY) has been prepared via a range of synthetic approaches using the diDABCO-C8 template, which has been reported previously. These include hydrothermal conversion, with or without preaging of a clear aluminosilicate gel before addition of crystallization organics, and partial IZC. The preaged gel and partial IZC accelerate the crystallization compared to the one-step hydrothermal synthesis due to the presence of preformed nuclei. All hydrothermal-aged gel conversions give crystals with “rice grain” morphology, with incomplete development of crystal facets, while the one using only diDABCO-C8, without TPA as a mineralizer, gives smaller crystals with high external area. Partial IZC gives “matchstick-like” crystals. By contrast with these, the aged gel approach using diQuin-C8 gives well-faceted crystals that reflect the crystal structure’s symmetry. All STA-30 materials have similar Si/Al ratios, from 6–7, enabling a ready comparison of their properties.

In the activated K,H-form, all STA-30 samples are highly crystalline and indistinguishable by PXRD. In their dehydrated forms, ^27^Al MAS NMR of all samples indicates at least two different octahedral Al species in addition to the majority of tetrahedral species: the former are attributed to octahedral framework-associated species. Adsorption studies with Ar, however, show strong differences. Those prepared using diDABCO-C8 as OSDA possess higher micropore volumes (by up to 30%) compared to that made with diQuin-C8. The latter’s pore volume of 0.24 cm^3^ g^–1^ is in line with that expected for the uptake of the ideal SWY topology and very similar to that observed for the closely related small-pore zeolite erionite. The extra porosity of the diDABCO-C8 materials is due to extra-large micropores ca. 13 Å in diameter that are present in addition to the *swy* and *gme* cages, which give the main peak in the pore distribution. ^1^H NMR and FTIR of dehydrated activated STA-30 materials consistently reveal very high silanol contents in the diDABCO-C8 samples and a low silanol content in the diQuin-C8 material, with lower and higher BAS concentrations, respectively. The defect silanol levels of STA-30 are therefore closely related to the presence of the “noncrystallographic” extra-large micropores.

Isopentane adsorption and diDABCO-C8^2+^ back exchange into activated STA-30 demonstrates that the extra-large micropores of diDABCO-C8-templated materials are connected to each other and to the exterior surface via windows at least 6 Å in diameter, while there are few if any pores accessible to these larger molecules in the diQuin-C8 material, which approximates to an “ideal” SWY zeolite. Additionally, the IR of adsorbed basic probe molecules pyridine and 2,4,6-collidine indicates that the pore structure is extra-large micropore/small micropore hierarchical because there is a high degree of accessibility of BAS associated with the SWY structure to the pyridine species that are too large to pass through 8Rs.

We have established a structural model for the additional extra-large micropores present in diDABCO-C8_STA-30, whose narrow pore-size distribution suggests that they result from a missing structural subunit. Remarkable STEM images confirm that the extra porosity arises from *can/d6r* column vacancies. As well as explaining the Ar adsorption isotherms and the IR data on the accessibility of BASs to pyridines, it resolves some hitherto puzzling features of diDABCO-C8_STA-30. These include the high organic content of the as-prepared zeolite and splittings in resonances in the ^13^C NMR of the included OSDA (not observed for diQuin-C8 STA-30) as well as the high silanol content of the activated material and its high specific N_2_ uptake compared with the similar K,H-erionite.

We speculate that this additional porosity could result from interruption of the growing *can*/*d6r* columns by H-bonding of terminal silanols with tertiary amine groups on the DABCO units and bridging of the pores by the diDABCO-C8 molecules. Notwithstanding the details, the data presented here show that the amount of this extra-porosity varies between preparations using the diDABCO-C8 template and so can be tuned by changes in the Al source and preparation route. Further, changing the template can greatly reduce or even eliminate this feature.

The preparation of hierarchical 3D extra-large micropore/small-pore STA-30 by direct synthesis and calcination represents a new approach of introducing a secondary type of “noncrystallographic” porosity in a small pore zeolite. This is advantageous because many of the current approaches for the introduction of additional porosity in zeolite make use of complex organic compounds or harsh processes that increase the cost of the preparation of the material and strongly reduce the yield. As observed for hierarchical mesoporous zeolites, this new feature in STA-30 could lead to enhanced diffusivity, for example, when used as a catalyst in the MTO reaction, for which the related erionite has been shown to be active and selective.

The larger amount of silanols introduced along with the secondary microporosity could prove useful in various catalytic reactions and also as sites for further functionalization, and the extra-large pores could provide sites for conversions of molecules up to 13 Å in dimension. Also, the more closely ideal small-pore SWY can be prepared by changing the OSDA, with a strongly reduced amount of silanols. Therefore, this work establishes routes for the synthesis of STA-30 with a tunable porosity and acidity. Work is ongoing to investigate whether the approach can be extended to other small pore zeolites.

## Supporting Information Available

Synthesis and characterization of OSDA; gel compositions, reagent sources, synthesis conditions, and products; additional zeolite syntheses and characterization for comparative materials; crystallization kinetics; TGA data and CHN analysis; comparative Ar adsorption isotherms; XRF and ^29^Si and ^27^Al MAS NMR; FTIR spectra of SWY, ERI, and OFF aluminosilicates and of their interaction with pyridine and collidine; additional ^13^C CP MAS NMR spectra; calculations of number of missing *can/d6r* columns; additional STEM-ADF data; and additional structural models (PDF)
