# Supplementary material for: Synthetic Control of the Defect Structure and Hierarchical Extra-Large-/Small-Pore Microporosity in Aluminosilicate Zeolite SWY
Source: J Am Chem Soc. 2023 Sep 27;145(40):22097–114. doi: 10.1021/jacs.3c07873 (PMC10571081; doi:10.1021/jacs.3c07873)
Supplement: Supplementary file 1 — ja3c07873_si_001.pdf [file ja3c07873_si_001.pdf]

Synthetic Control of Defect Structure and Hierarchical Extra-Large-/Small-Pore Microporosity in Aluminosilicate Zeolite SWY

Ruxandra G. Chitac,[1] Vladimir L. Zholobenko,[2,**] Robin S. Fletcher,[3] Emma Softley,[3] Jonathan Bradley,[4] Alvaro Mayoral,[5] Alessandro Turrina[6,*] and Paul A. Wright[1,*]

[1] EaStCHEM School of Chemistry, University of St Andrews, KY16 9ST, U.K.

[2] School of Chemical and Physical Sciences, Keele University, Staffordshire, ST5 5BG, U.K.

[3] Johnson Matthey, Catalyst Technologies, Billingham, TS23 1LB, U.K.

[4] Johnson Matthey Technology Centre, Sonning Common, RG4 9NH, U.K.

[5] Instituto de Nanociencia y Materiales de Aragon (INMA), Spanish National Research Council (CSIC)-University of Zaragoza 12, Calle de Pedro Cerbuna, 50009, Zaragoza, Spain;

[6] Johnson Matthey Technology Centre, Chilton, TS23 1LB, U.K.

* *Corresponding authors:* alessandro.turrina@matthey.com, paw2@st–andrews.ac.uk.

** Current Addresses: Department of Chemistry, Moscow State University, Moscow, 119991, Russian Federation; School of Chemical and Physical Sciences, Keele University, Staffordshire, ST5 5BG, U.K.

Contents

Synthesis and characterisation of 1,8–(1–azabicyclo[2.2.2]octane)octyl dibromide – (diQuin-C8)Br_2 2

Partial interzeolite conversion synthesis product without the use of seeds 3

Gel compositions, reagent sources and synthesis conditions 3

ERI/OFF/STA-30 comparison 5

Synthesis of aluminosilicate ERI sample 5

Synthesis of aluminosilicate OFF sample 5

Crystallisation kinetics 7

Synthesis gels 7

^{13}C NMR of OSDA solutions 8

TGA data 8

CHN analysis results 9

Ar adsorption isotherms at 87 K of SWY, ERI and OFF aluminosilicate materials 9

Silica to Alumina ratio (SAR) by XRF and NMR.. 9

Additional FTIR spectra...10

FTIR spectra of SWY, ERI and OFF aluminosilicate materials 10

^{13}C CP–MAS NMR spectrum of back-exchanged diDABCO-C8_STA-30 12

Frozen isopentane Ar adsorption experiment 12

Calculations of number of missing *can/d6r* columns 13

FTIR spectra of the interaction of TPA_AliPr_H with pyridine and collidine...........................13

Additional STEM–ADF data...15

ITQ-43 structure vs STA-30 with extra-large micropores 17

References...18

Synthesis and characterization of 1,8–(1–azabicyclo[2.2.2]octane)octyl dibromide – (diQuin-C8)Br_2

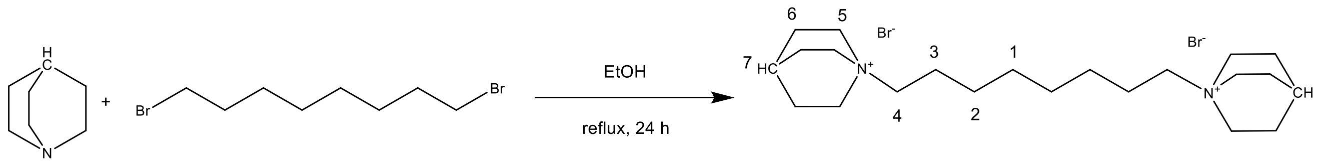

7.1 g (63.8 mmol) quinuclidine (TCI) and 9.2 g (33.1 mmol) 1,8–dibromooctane (Lancaster Chemicals) were dissolved in 120 mL ethanol. The mixture was heated under reflux for 24 hours. The solvent was removed with a rotary evaporator and the resulting white solid was washed with cold diethyl ether and acetone. The resulting white powder (15.6 g, 99% yield) was dried overnight and then analysed by NMR.

^{1}H NMR: δ_H (400 MHz, D_2O) 1.24–1.27 (8 H, m, H1+H2), 1.63 (4 H, m, H3), 1.90 (12 H, m, H6), 2.10 (2 H, m, H1), 3.02 (4 H, m, H4), 3.30 (12 H, t, H5). **^{13}C NMR:** δ_C (100 MHz, D_2O) 19.1 (C7), 21.4 (C3), 23.4 (C6), 25.6 (C6), 27.9 (C7), 54.6 (C5), 64.3 (C4).

Partial interzeolite conversion synthesis product without the use of seeds

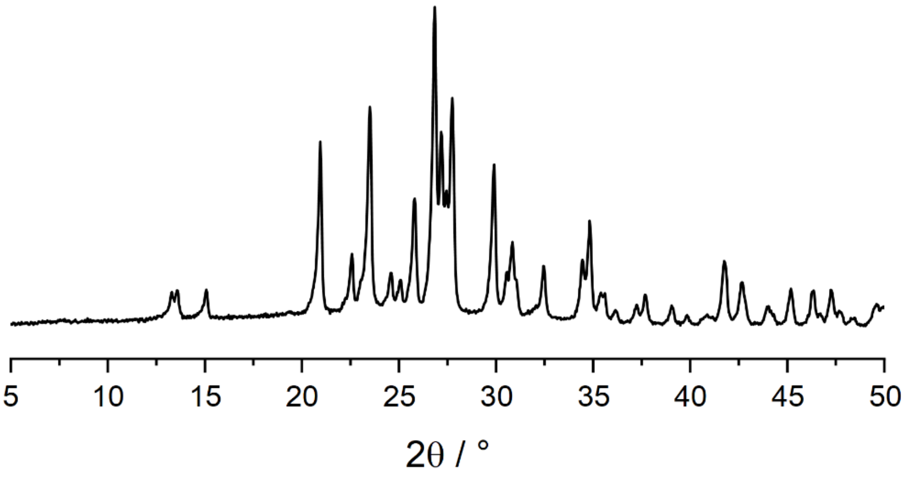

Figure S1. PXRD pattern of the product of the seed–free partial interzeolite conversion gel composition used for the synthesis of STA-30.

Gel compositions, reagent sources and synthesis conditions

Table S1 Gel components and molar gel composition

**Sample code**	**Mineraliser source**	**Al source**	**Si Source**	**Molar ratio to SiO_2 in the gel**[a]					**Seeds**	**Yield / %**
				**Mineraliser**	**Al_2O_3**	**KOH**	**(OSDA)Br_2**[b]	**H_2O**		
TPA_AliPr	TPA^+	Al(O–i–Pr)$_3$	Ludox HS–40	0.4	0.04	0.09	0.06	20	–	100
TPA_AlOH	TPA^+	Al(OH)$_3$	Ludox HS–40	0.4	0.04	0.08	0.13	20	–	48
OSDA_AlOH	diDABCO-$C8^{2+}$	Al(OH)$_3$	Ludox HS–40	0.2	0.06	0.08	0.07	25	–	77
OSDA_Y	diDABCO-$C8^{2+}$	CBV 712	CBV 712 + Ludox HS–40	0.2	0.06	0.23	0.07	21	7.5%[d]	75
TPA_diQuin-C8	TPA^+	Al(O–i–Pr)$_3$	Ludox HS–40	0.4	0.05	0.19	0.16[c]	20	–	100

[a] All molar quantities as ratio to 1.0 SiO_2. [b] OSDA was diDABCO-$C8^{2+}$, unless specified otherwise. [c] OSDA used for this sample was diQuin-$C8^{2+}$. [d] TPA_AliPr or TPA_AlOH type samples were used as seeds. Weight percent calculated in respect to SiO_2 content.

Table S2 Reagents sources

**Chemical**	**Formula used in text**	**Purity/ Concentration**	**Source**
Tetrapropylammonium hydroxide	TPAOH	40 wt.% aq. sol.	SACHEM
1,8–(1,4–diazabicyclo [2.2.2]octane)octyl hydroxide	(diDABCO-C8)$(OH)_2$	20 wt.% aq. sol.	Alfa Aesar
Aluminium isoproxide	Al(O–*i*–Pr)$_3$	98%	Sigma Aldrich or Acros Organics
Aluminium hydroxide	$Al(OH)_3$	87.8%	Alfa Aesar
CBV 712	CBV 712	SAR 12	Zeolyst
Ludox HS–40 colloidal silica	Ludox HS–40	40 wt.% suspension in H_2O	Sigma Aldrich
1,8–(1,4–diazabicyclo [2.2.2]octane)octyl bromide	(diDABCO-C8)Br_2	100%	Alfa Aesar
1,8–(1–azabicyclo [2.2.2]octane)octyl dibromide	(diQuin-C8)Br_2	100%	In–house
Potassium hydroxide	KOH	85%	Alfa Aesar or Fisher Scientific

Table S3 Synthesis conditions

**Sample code**	**Scale / mL**	**Temperature / °C**	**Time / days**	**Aging before SDAs**	**Mixing during heating**
TPA_AliPr	1500	135	3	Yes	Mechanical stirring, 300 rpm
TPA_AlOH	1500	135	5	Yes	Mechanical stirring, 300 rpm
OSDA_AlOH	1500	135	6	No	Mechanical stirring, 300 rpm
OSDA_Y	125	160	2	No	Rotation, 60 rpm
TPA_diQuin-C8	50	135	7	No	Rotation, 60 rpm

ERI/OFF/STA-30 comparison

Synthesis of aluminosilicate ERI sample

The synthesis was designed based on the work published by Lee *et al.*[2]

In a Teflon liner, aluminium isopropoxide was dissolved in TPAOH aq. sol. 40% by stirring for at least 30 min at RT. Ludox HS–40 was then added to this solution and the resulting gel was allowed to stir for 1.5 h at RT. The gel was aged at 368 K for 20 h before the addition of the SDAs. KOH was dissolved in DI H_2O and added dropwise to the gel while stirring. Then, 1–methylpyrroldine and 1,4–dibromobutane were added. The final gel composition was 0.04 Al_2O_3 : 1.0 SiO_2 : 0.54 TPAOH : 0.13 KOH : 0.1 1,4–dibromobutane : 0.2 1–methylpyrrolidine : 19.3 H_2O. The gel was allowed to stir for minimum 2 h, after which it was loaded into a stainless–steel autoclave and heated under tumbling at 60 rpm for 5 days at 423 K. The resulting solid was separated by centrifugation at 14500 rpm and washed with DI H_2O until a neutral pH was achieved. The solid was dried in a 338 K oven overnight. The Si/Al of the product, as characterised by EDS was 6.1.

Synthesis of aluminosilicate OFF sample

The synthesis was based on the work carried out by Łukaszuk *et al.*[3]

Sodium hydroxide, potassium hydroxide and water were mixed and then heated up to 348 K. Aluminium isopropoxide was added and the mixture was stirred for 15 min after which the heating was stopped. The solution was added to Ludox HS–40, and the mixture was left to stir for minimum 30 min. TMACl was then added and the mixture was allowed to stir for a final hour. The gel composition was 0.04 Al_2O_3 : 1.0 SiO_2 : 0.57 NaOH : 0.14 KOH : 0.02 TMACl : 11.3 H_2O. The gel was transferred into a stainless–steel autoclave equipped with a Teflon liner and heated for 8 days at 373 K. The resulting solid was filtered and washed with DI water until the filtrate had a neutral pH. The solid was dried in a 338 K oven overnight. The Si/Al of the product, as characterised by EDS was 3.3.

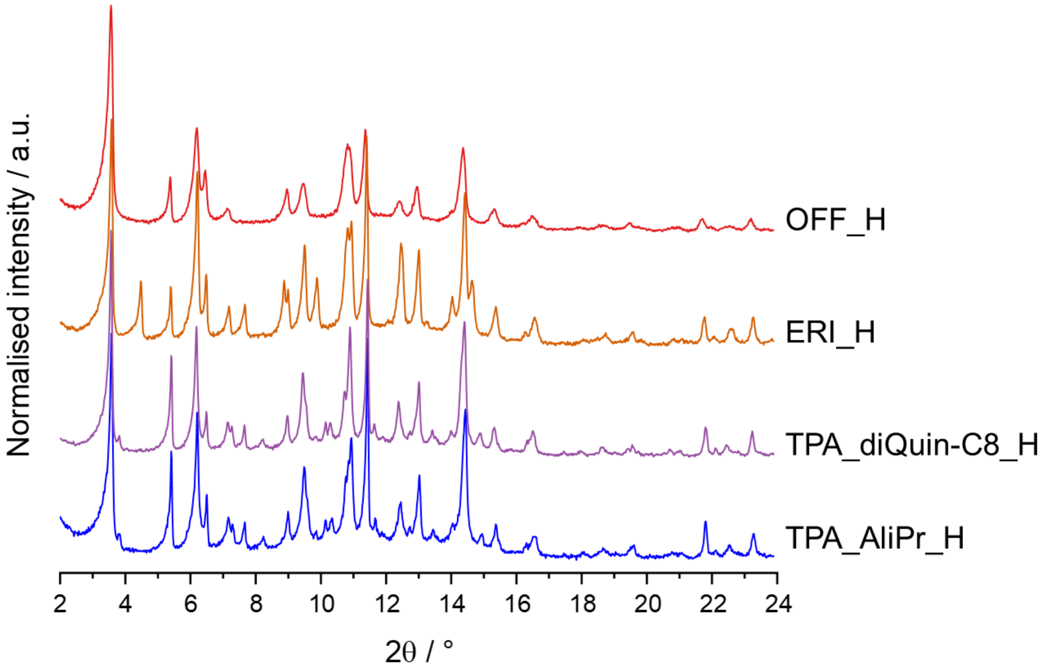

Figure S2. PXRD patterns collected with Mo $K_{\alpha1}$ X-radiation of H–forms of TPA_AliPr, TPA_diQuin-C8, ERI and OFF.

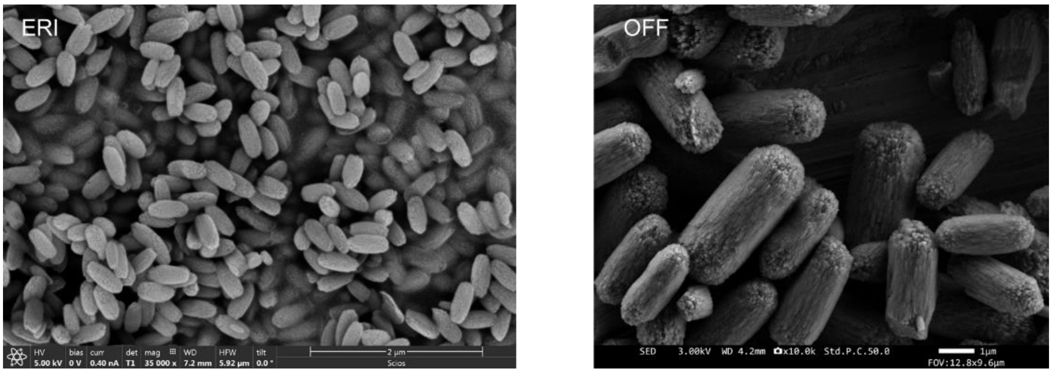

Figure S3. SEM images of aluminosilicate ERI and OFF described above.

Crystallization kinetics

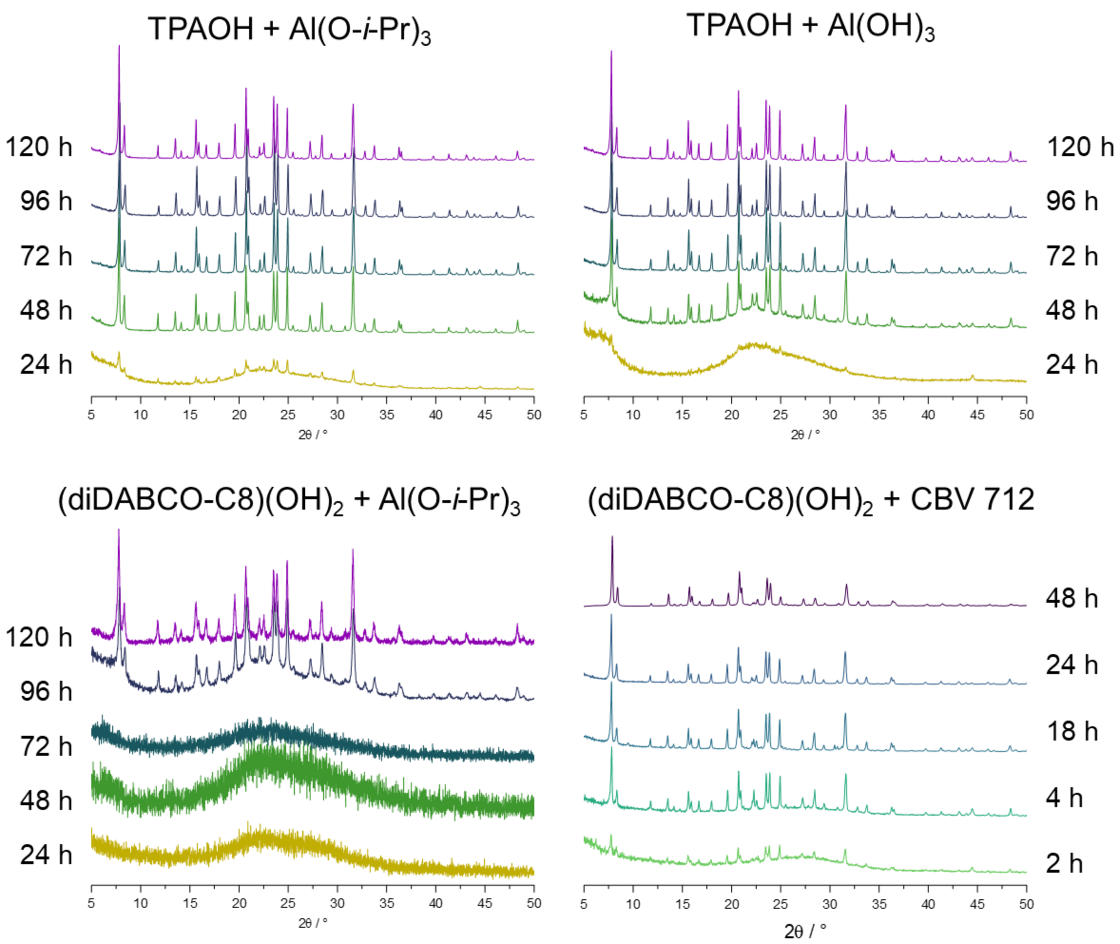

Figure S4. PXRD patterns of solids recovered after varying amounts of time from different synthetic routes to STA-30.

Synthesis gels

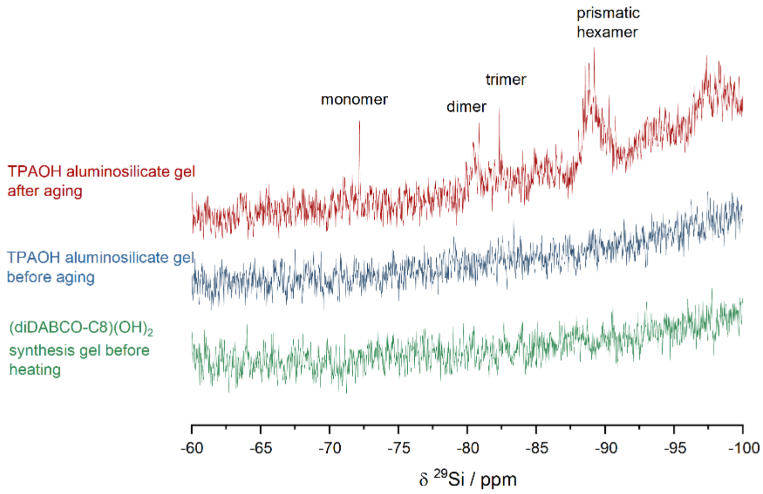

Figure S5. ^{29}Si NMR spectra of synthesis gels with assignments based on Cho *et al.*[1]

^{13}C NMR of OSDA solutions

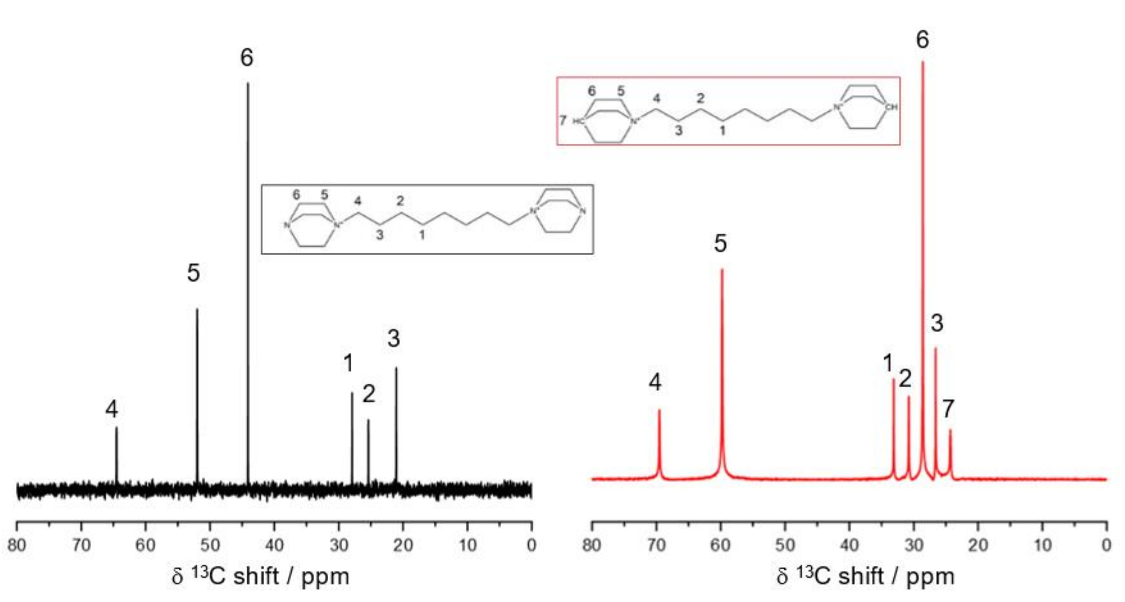

Figure S6. ^{13}C NMR spectra of (diDABCO-C8)Br_2 in D_2O (left) and (diQuin-C8)Br_2 in D_2O (right).

TGA data

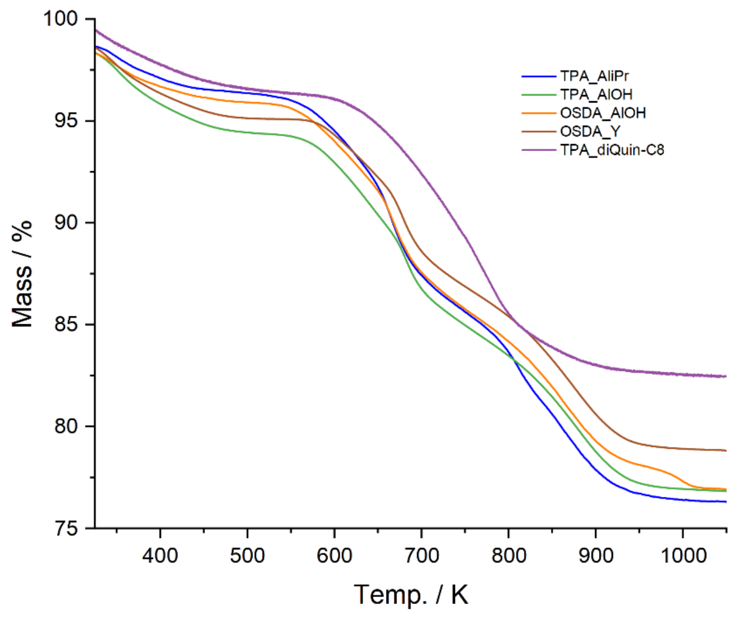

Figure S7. TGA mass loss curves of TPA_AliPr (blue), TPA_AlOH (green), OSDA_AlOH (orange), OSDA_Y (brown) and TPA_diQuin-C8 (purple).

CHN analysis results

Table S4 CHN analysis results and comparison with expected values.

Sample	%C	%H	%N	C/N	OSDA formula	Expected C/N
diQuin-C8-STA-30	10.2	1.89	1.06	11.2	$C_{22}H_{42}N_2^{2+}$	11
diDABCO-C8-STA-30	12.8	2.43	2.78	5.4	$C_{20}H_{40}N_4^{2+}$	5

Ar adsorption isotherms at 87 K of SWY, ERI and OFF aluminosilicate materials

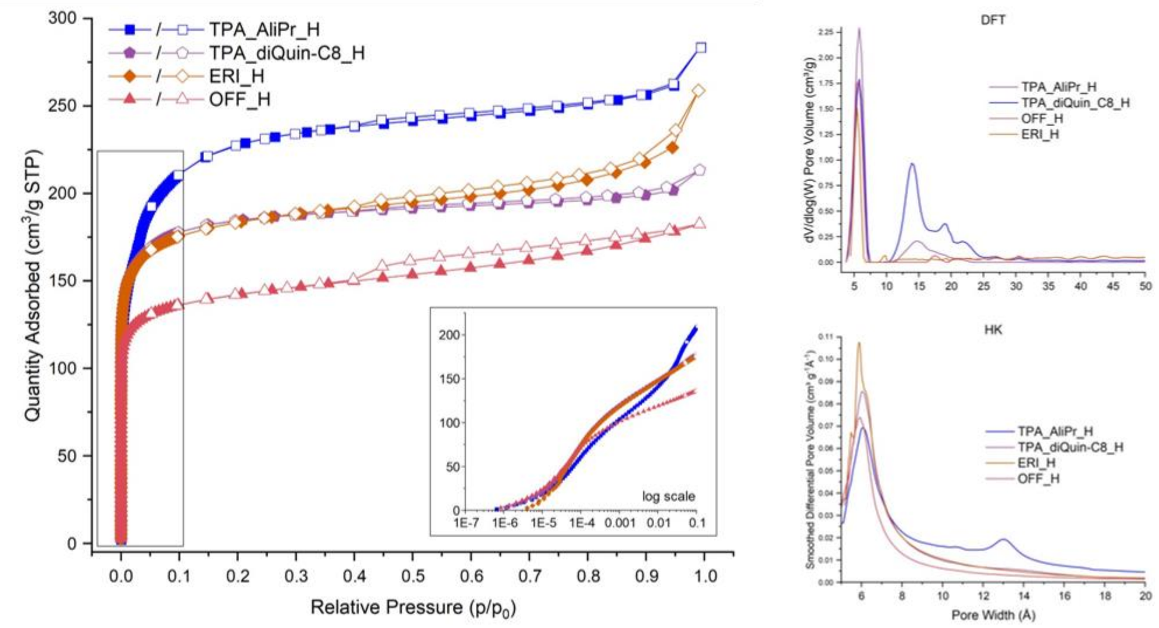

Figure S8. Ar adsorption/desorption isotherms measured at 87 K on the H–forms of TPA_AliPr, TPA_diQuin-C8, ERI and OFF (left, insert shows an enlarged view of 10^{-7}–0.1 p/p_o). The DFT and HK pore size distributions are also plotted (top right and bottom right).

Silica to Alumina ratio (SAR) by XRF and NMR

Table S5 Comparison of silica–to–alumina ratio (SAR) in the products as determined by XRF or ^{29}Si MAS NMR

Sample	XRF SAR	NMR SAR
**TPA_AliPr_H**	12.0	13 ± 1
**TPA_AlOH_H**	14.1	15 ± 1
**OSDA_AlOH_H**	13.5	15 ± 1
**OSDA_Y_H**	12.0	13 ± 1
**TPA_diQuin-C8_H**	11.9	11.9 ± 0.7

Additional FTIR spectra

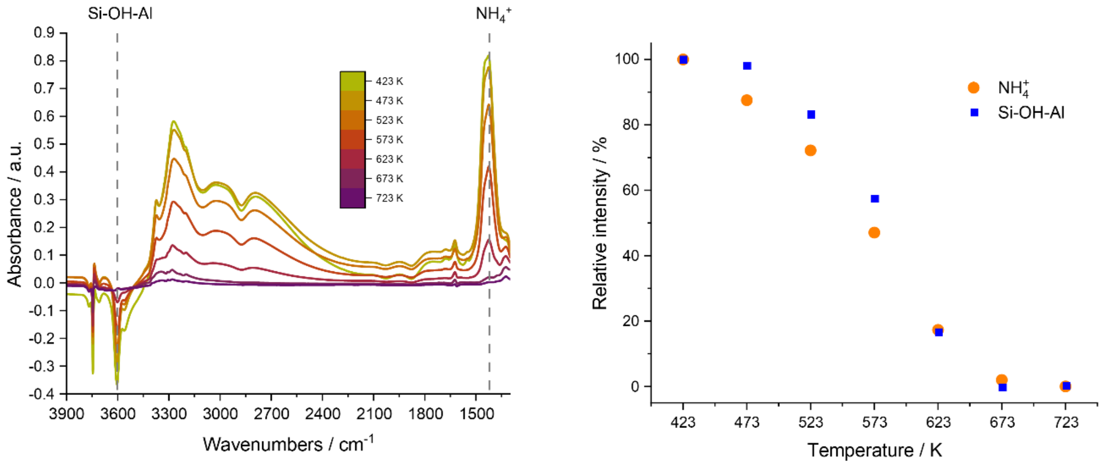

Figure S9. Difference FTIR spectra between the spectrum of dehydrated TPA_AliPr_H and spectra collected at temperatures between 423 K and 723 K after the structure had been loaded with NH_3 (left) and the variation of the intensity of the peaks that show the interaction between NH_3 and the zeolite acid sites.

FTIR spectra of SWY, ERI and OFF aluminosilicate materials

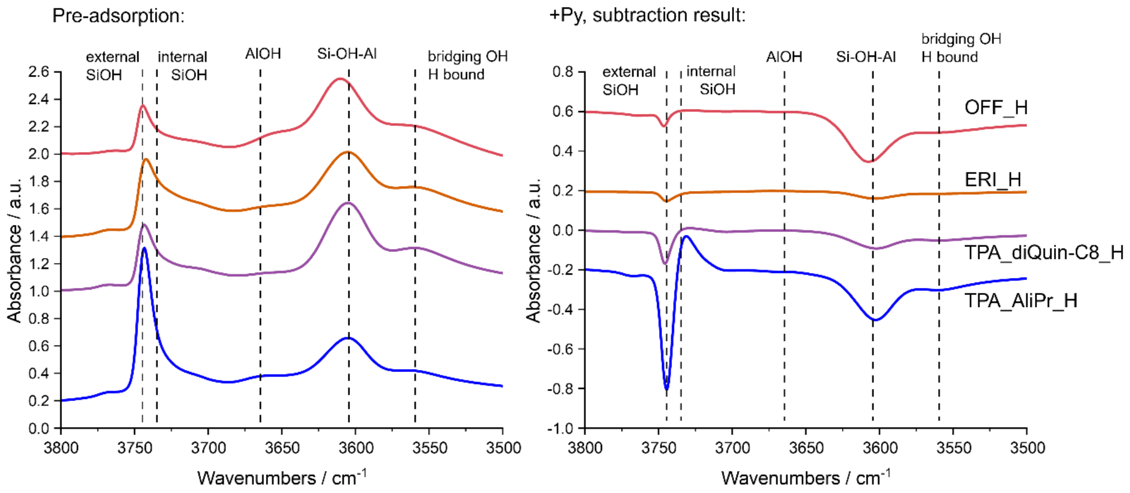

Figure S10. SiOH region of the FTIR spectra of the H–forms of TPA_AliPr, TPA_diQuin-C8, ERI and OFF, after dehydration (left) and difference FTIR spectra between the aforementioned data and the spectra of the samples upon pyridine (Py) adsorption at 473 K (right). Absorbance values were normalized, and spectra were offset for ease of visualisation.

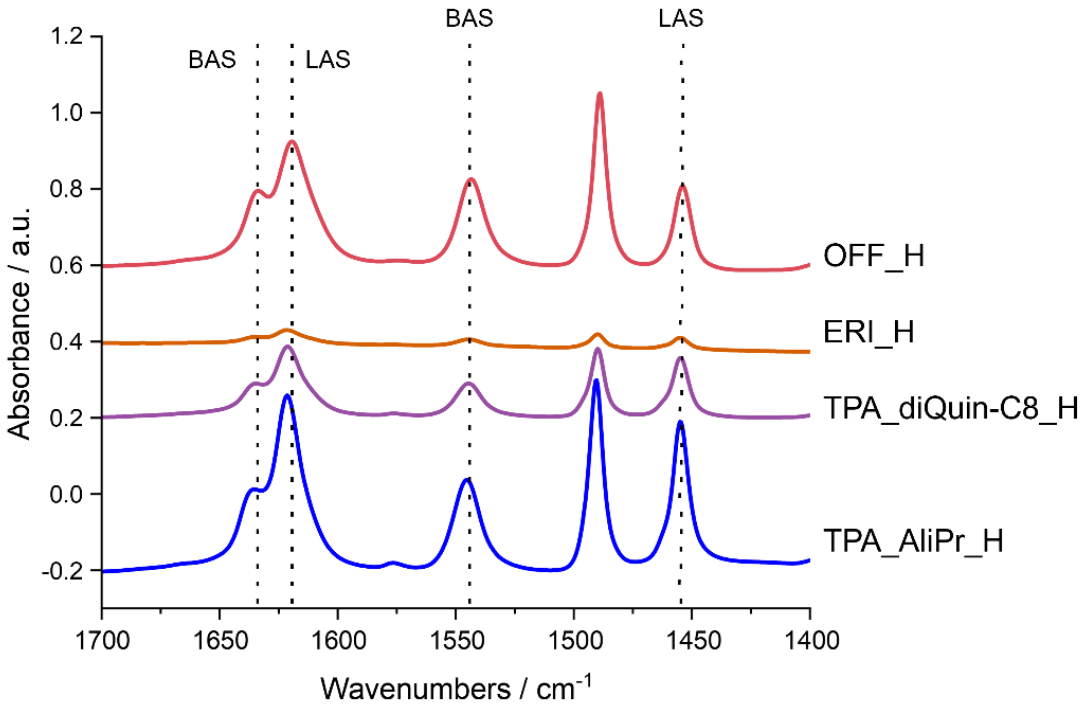

Figure S11. Py region of the difference FTIR spectra between the spectra of the dehydrated samples (H–forms of TPA_AliPr, TPA_diQuin-C8, ERI and OFF) and the spectra of the samples upon pyridine adsorption at 473 K. Absorbance values were normalised and spectra were offset for ease of visualisation.

^{13}C CP–MAS NMR spectrum of back-exchanged diDABCO-C8_STA-30

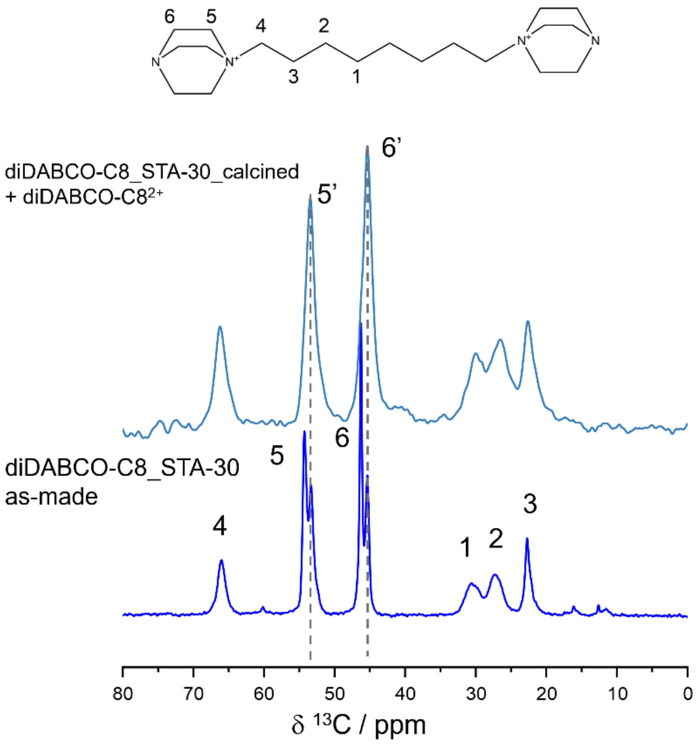

Figure S12. ^{13}C CP–MAS NMR spectra of diDABCO-C8_STA-30 as-made (bottom, blue) and the calcined sample of diDABCO-C8_STA-30 that had been back-exchanged with a solution of (diDABCO-C8)Br_2 (top, light blue).

Frozen isopentane Ar adsorption experiment

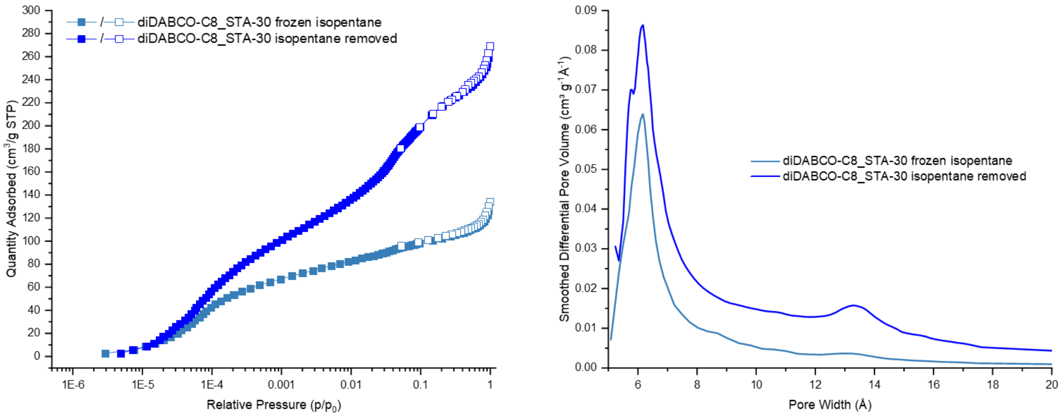

Figure S13. Ar adsorption isotherms at 87 K (left) and derived HK plots (right) collected after pre-adsorbed isopentane was frozen into pores (light blue) and after the evacuation of isopentane and Ar (blue). The isotherms are plotted on a logarithmic scale of p/p_0.

Calculations of number of missing *can/d6r* columns

$$Uptake\ (cm^3\ g^{-1}\ STP) = \frac{\frac{no.of\ Ar\ atoms/unit\ cell}{N_A} \cdot V_{m\ (STP)}}{\frac{M_{unit\ cell}}{N_A}} = \frac{no.of\ Ar\ atoms/unit\ cell \cdot 22.4 \cdot 10^3}{M_{unit\ cell}}$$

Ar adsorption isotherm simulation performed on ideal SWY unit cell with K occupying all *can* cages which had the formula $K_4Si_{72}O_{144} \Rightarrow M_{unit\ cell\ ideal}$ = 4482.46 g mol^{-1}. At 50 kPa, the number of Ar atoms adsorbed into the unit cell according to the Sorption calculation was 39.7. Thus, by applying the formula above, the uptake of the ideal unit cell would be 198.4 cm^3 g^{-1}.

Ar adsorption isotherm simulation performed on 2×2×1 SWY supercell that had a column of *can* cages and d6r removed, H added for any Si–O terminations introduced through the removal and K positioned into all other *can* cages. This supercell had the formula $H_{24}K_{12}Si_{216}O_{444} \Rightarrow M_{supercell\ with\ vacancies}$ = 13663.57 g mol^{-1}. At 50 kPa, the number of Ar atoms adsorbed into this supercell according to the Sorption calculation was 208.3. Thus, by applying the formula above, the uptake of this supercell would be 341.5 cm^3 g^{-1}.

For a sample that is theoretically made up of a fraction of ideal SWY unit cells (considered below as an ideal 2×2×1 SWY supercell) and a fraction, $f_{vacancies}$, of 2×2×1 SWY supercells with removed *can* columns, the total uptake is made up of:

$$Total\ experimental\ uptake = \frac{f_{ideal} \cdot 198.4 \cdot M_{ideal} + f_{vacancies} \cdot 341.5 \cdot M_{supercell\ with\ vacancies}}{M_{dehydrated\ zeolite}},$$

where $f_{ideal} + f_{vacancies} = 1$,

$$M_{dehydrated\ zeolite} = f_{ideal} \cdot M_{ideal} + f_{vacancies} \cdot M_{supercell\ with\ vacancies}\ and$$

$$M_{ideal} = 17929.84\ g\ mol^{-1} and\ M_{supercell\ with\ vacancies} = 13663.57\ g\ \ mol^{-1}$$

The experimental uptake at 0.5 relative pressure for TPA_AliPr_H was 241.4 cm^3 g^{-1}. By using this number in the relationship above, the fraction of ideal unit cells and 2×2×1 supercells with vacancies are:

$$f_{ideal} = 0.64 \text{ and } f_{vacancies} = 0.36.$$

If ~36% of the structure of TPA_AliPr_H is described by the 2×2×1 supercell with a column of *can*/d6r cages removed and 3 columns intact, and the rest of the structure is described by the ideal unit cell, this means that ~9% of *can/d6r* cages have to be removed to introduce the additional porosity observed in TPA_AliPr_H.

The experimental uptake at 0.5 relative pressure for OSDA_Y_H was 212.3 cm^3 g^{-1}. By plugging this number into the total uptake equation, the fraction of ideal unit cells and 2×2×1 supercells with vacancies are:

$$f_{ideal} = 0.88 \text{ and } f_{vacancies} = 0.12.$$

If ~12% of the structure of OSDA_Y_H is described by the 2×2×1 supercell with a column of *can*/d6r cages removed and 3 columns intact, and the rest of the structure is described by the ideal unit cell, this means that ~3% of *can*/*d6r* cages have to be removed to introduce the additional porosity observed in OSDA_Y_H.

FTIR spectra of the interaction of TPA_AliPr_H with pyridine and collidine

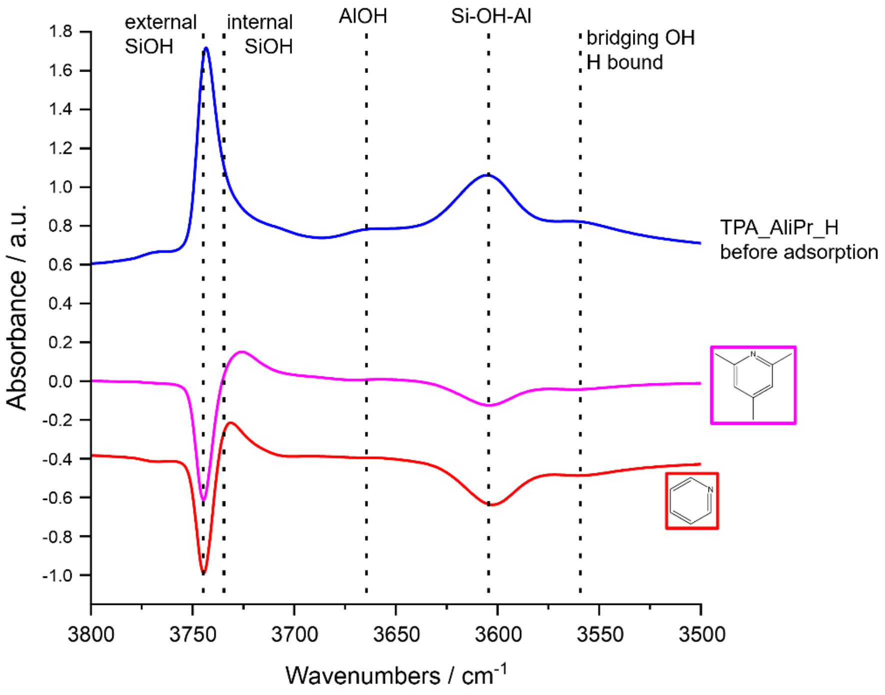

Figure S14. SiOH region of FTIR spectrum of TPA_AliPr_H dehydrated at 723 K and offset difference spectra showing the interaction between TPA_AliPr_H with pyridine (red outline) and collidine (pink outline). Absorbance values were normalized, and spectra were offset for ease of visualisation.

Additional STEM–ADF data

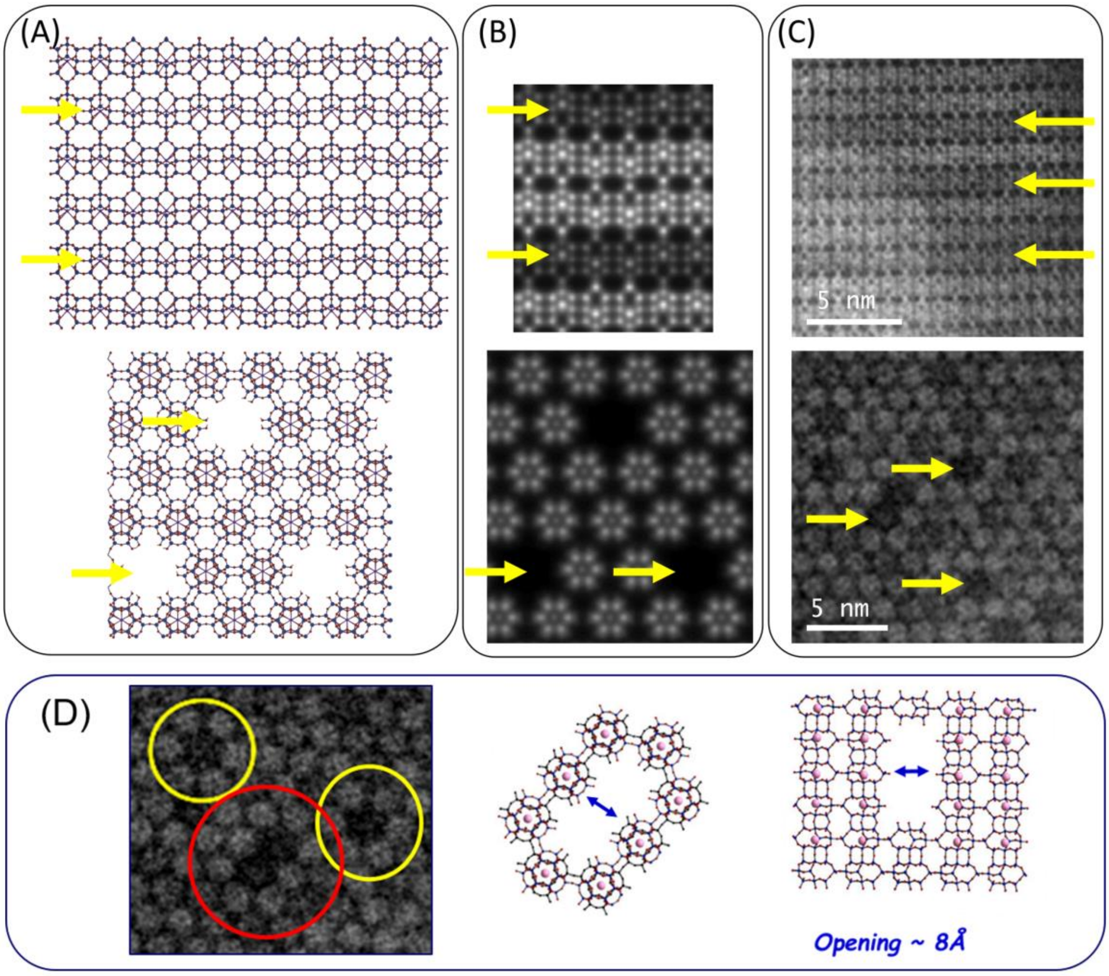

Figure S15. (A) Schematic models of STA-30 prepared with diDABCO-C8^{2+} observed along the [100] (top) and [001] (bottom) zone axes. The yellow arrows indicate where some *can/d6r* columns are missing. For clarity, along [001] a complete column was removed. Those missing units are not revealed when looking along [100] as some of the remaining units are observable along this orientation. However, in the simulated data in (B) the signal is affected by the missing columns. When some of the *can/d6r* columns are removed, the crystal is thinner as those regions and therefore the signal is lower, as indicated by yellow arrows (top image along [100]). This effect is more pronounced when the image was simulated along the [001] zone axis, where some of the columns are absent. In the experimental image (C), these effects are not as marked as in the simulated data as other parameters such as signal to noise ratio also influences the contrast. Nevertheless, in the bottom image of (C), the obvious existence of vacancies along [001] is indicated with yellow arrows. Along [100] the contrast difference is not as evident because along this projection the remaining units are also observed. Despite this, the contrast differences are also visible as indicated by yellow arrows. Considering that the composition is the same along the crystal, such contrast variation results from the absence of *can/d6r* columns. Note that for a clear visualization, complete *can/d6r* columns have been removed in the model; experimentally, it is unlikely that this is the case and some of the *can/d6r* units may remain in the individual columns. This is what gives rise to a weak signal visible in the columns indicated by yellow arrows. In (D) a double defect seen in the STEM image is marked by a red circle. Between these

neighbouring missing columns there would be slit pores ca. 8 Å across (allowing for van der Waals radii of atoms) illustrated schematically in the middle and right figures.

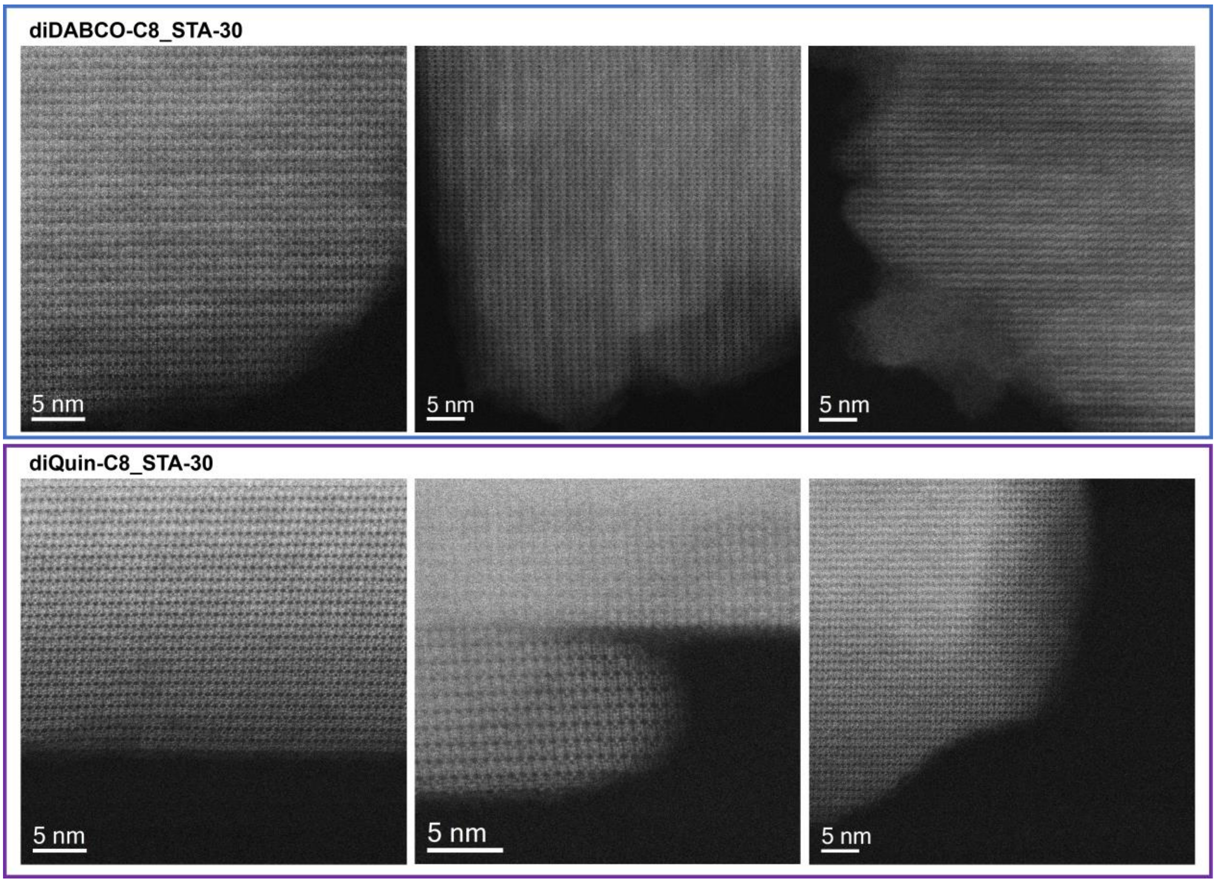

Figure S16. Additional C_s-corrected STEM-ADF images collected from diDABCO-C8_STA-30 (top) and diQuin-C8_STA-30 (bottom). The figures were not annotated, but it is clear that the top figures all show differences in contrast among certain *can/d6r* columns, whereas the columns in diQuin-C8_STA-30 do not show this feature.

ITQ-43 structure vs STA-30 with extra-large micropores

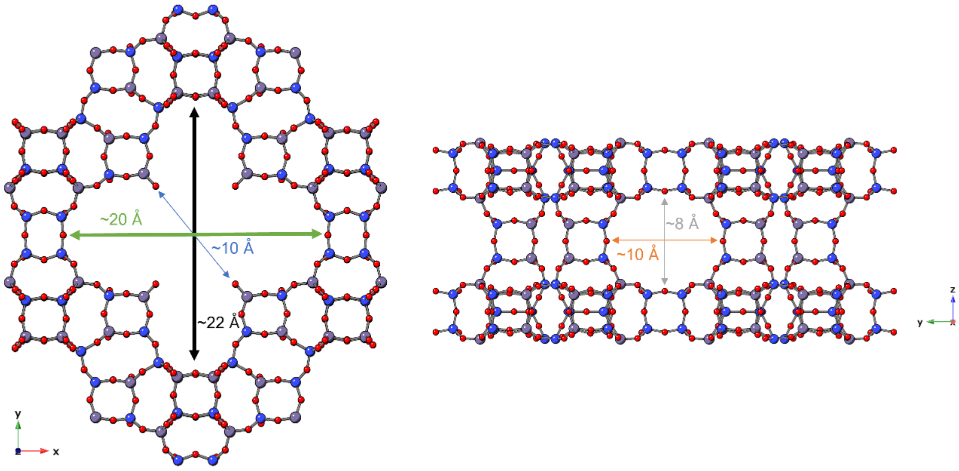

Figure S17. Structure of germanosilicate ITQ-43, downloaded from the IZA database and edited in CrystalMaker.[4,5]

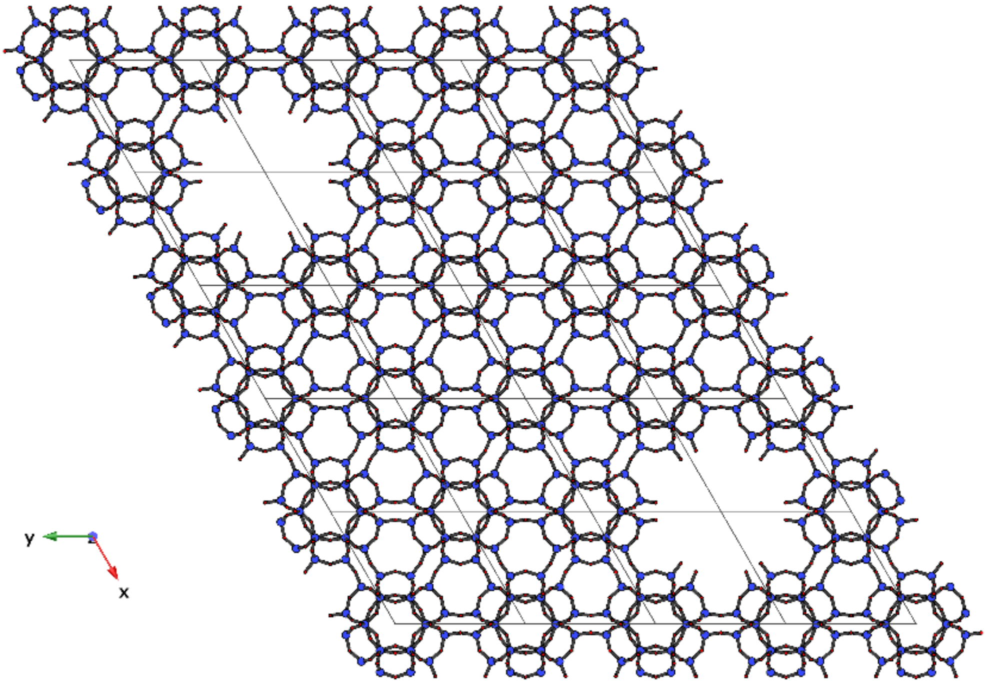

Figure S18. Schematic portrayal of 1 in 10 columns of *can+d6r* columns missing from SWY topology.

References

(1) Cho, H.; Felmy, A. R.; Craciun, R.; Keenum, J. P.; Shah, N.; Dixon, D. A. Solution State Structure Determination of Silicate Oligomers by ^{29}Si NMR Spectroscopy and Molecular Modeling. *J. Am. Chem. Soc.* **2006**, *128*, 2324–2335.

(2) Lee, J. H.; Park, M. B.; Lee, J. K.; Min, H.-K.; Song, M. K.; Hong, S. B. Synthesis and Characterization of ERI-Type UZM-12 Zeolites and Their Methanol-to-Olefin Performance. *J. Am. Chem. Soc.* **2010**, *132*, 12971–12982.

(3) Łukaszuk, K. A.; Rojo-Gama, D.; Øien-Ødegaard, S.; Lazzarini, A.; Berlier, G.; Bordiga, S.; Lillerud, K. P.; Olsbye, U.; Beato, P.; Lundegaard, L. F.; Svelle, S. Zeolite Morphology and Catalyst Performance: Conversion of Methanol to Hydrocarbons over Offretite. *Catal. Sci. Technol.* **2017**, *7*, 5435–5447.

(4) IZA database http://www.iza-structure.org/databases/ (accessed Jun 30, 2023).

(5) CrystalMaker Software, Oxford 2023.
